# Prins fluorination cyclisations: Preparation of 4-fluoro-pyran and -piperidine heterocycles

**DOI:** 10.3762/bjoc.6.41

**Published:** 2010-04-26

**Authors:** Guillaume G Launay, Alexandra M Z Slawin, David O'Hagan

**Affiliations:** 1 School of Chemistry and Centre for Biomolecular Sciences, University of St Andrews, North Haugh, St Andrews, Fife, KY16 9ST, United Kingdom

**Keywords:** 4-fluoropiperidine, 4-fluoropyran, heterocycles, organo-fluorine chemistry, Prins cyclisation

## Abstract

The Prins reaction was investigated using BF_3_·OEt_2_ as a Lewis acid. It has been recently demonstrated, that if BF_3_·OEt_2_ is used in stoichiometric amounts then these reactions generate fluorinated products where the BF_3_·OEt_2_ contributes fluoride ion to quench the intermediate carbocations. In this study oxa- and aza-Prins reactions for the synthesis of 4-fluoro-pyrans and -piperidines were investigated. The products were obtained in good yields, but only with moderate diastereoselectivity. These Prins fluorination reactions can be accelerated under microwave conditions. The study extends the Prins fluorination methodology for the generation of the C–F bond in heterocycles.

## Introduction

**Scheme 1:**
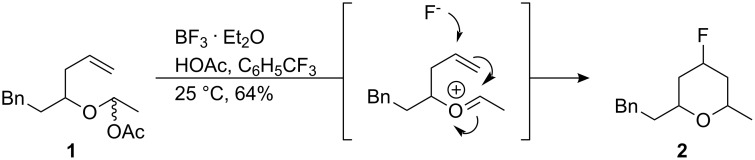
The C–F bond forming Prins reaction leading to 4-fluoropyrans [10].

Selective incorporation of the C–F bond into organic molecules can impart useful and attractive properties to performance materials [1–3]. To this end there are a useful but relatively limited range of fluorination reagents and methodologies available to synthetic organic chemistry, and novel methods for introducing fluorine into organic molecules continue to be valuable [4]. In this paper we focus on extending the scope of the Prins fluorination reaction as a synthetic methodology. The Prins reaction is a well established strategy for the synthesis of pyrans [5–7]. This cyclisation reaction, which occurs between a homoallylic alcohol and an aldehyde, is generally promoted by a Lewis acid. When BF_3_·OEt_2_ is used as the Lewis acid, then fluoride ion from the reagent can become incorporated into the product generating a C–F bond and a new stereogenic centre. Liberation of fluoride ion from BF_3_·OEt_2_ has, for example, been observed in epoxide ring opening reactions [8–9]. This was first recognised in a Prins reaction as an unexpected side reaction by Al-Mutairi et al. [10–11] and was noted separately by Jaber et al. [12] and subsequently by Kataoka et al. [13]. For example, homoallylic alcohol **1** was converted to pyran **2** with a high diastereoselectivity (Scheme 1) [12]. Most recently, oxa-, aza- and thia-Prins fluorination cyclisations have been carried out using ionic liquid hydrogen fluoride salts (Et_4_NF·5HF) as the reaction medium, without the requirement for BF_3_·OEt_2_ [14–15]. These reactions with fluoride follow from the much more commonly observed Prins reactions of halides (Cl^−^, Br^−^ and I^−^) other than fluoride in the quenching of the intermediate oxonium intermediate [16–22].

We have explored C–F bond formation by the BF_3_·OEt_2_/Prins reaction further. In this paper we report that a wide range of 4-fluorotetrahydropyrans can be prepared by reaction of homoallylic alcohols with different aldehydes with BF_3_·OEt_2_ as the fluoride source. This Prins methodology was extended to the aza-Prins reaction using *N*-tosyl-homoallylic amines to generate the corresponding 4-fluoropyrrolidines. In general, these reactions leading to both the 4-fluorotetrahydropyrans and 4-fluoro-pyrrolidines occur with good to high conversions, however the diastereoselectivities are modest, particularly in the aza-Prins cases. This study also demonstrates that the conversions and reaction times can be improved using microwave conditions.

## Results and Discussion

The oxa-Prins fluorination reaction: Oxa-Prins fluorination reactions were investigated with but-3-en-1-ol (**3**) and a range of substituted benzaldehydes **4**. Electron withdrawing groups on the aromatic ring led to the more efficient reactions (Table 1, entries a–g) to generate 4-fluoropyrans **5** with conversions of 65–73%. Diastereoselectivities were however, modest with d.r’s of between 1.9 : 1 and 5.4 : 1. With electron donating groups on the aromatic ring (Table 1, entries h–j), the reactions were inefficient and conversions dropped dramatically. The saturated aliphatic aldehyde, hexanal (entry k), resulted in a good conversion, although the corresponding 4-fluoropyran products were obtained with poor diastereoselectivity (2 : 1). In the case of 2-methylcinnamaldehyde (entry l), the conversion was poor.

**Table 1:** Prins fluorination reaction of homoallylic alcohol (**3**) with various aldehydes **4** giving substituted fluoropyrans **5**. Reaction conditions: BF_3_·OEt_2_ (1 equiv), but-3-en-1-ol (1 equiv), aldehyde (1 equiv), DCM, rt, 2 h.

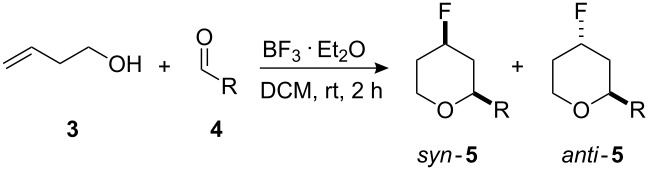			
pyran	aldehyde	conversion	d.r. (*syn/anti*)

**5a**	4-nitrobenzaldehyde	67%	1.9/1
**5b**	2-fluorobenzaldehyde	66%	4.5/1
**5c**	3-fluorobenzaldehyde	66%	3.4/1
**5d**	4-fluorobenzaldehyde	66%	4.5/1
**5e**	2-bromobenzaldehyde	65%	5.4/1
**5f**	3-bromobenzaldehyde	73%	3.8/1
**5g**	4-bromobenzaldehyde	90%	4.8/1
**5h**	2-methoxybenzaldehyde	<5%	1.3/1
**5i**	4-methoxybenzaldehyde	20%	2.4/1
**5j**	2,3,6-trimethoxybenz- aldehyde	no reaction	/
**5k**	hexanal	76%	2/1
**5l**	2-methylcinnamaldehyde	<5%	/

**Microwave – oxa-Prins:** The Prins fluorination reactions were then investigated under microwave conditions (Table 2 and Scheme 3). Reaction times were significantly reduced to 10 min and, in general, the conversions were higher than under the more classical conditions. The diastereoselectivity appears to decrease a little, and in some cases there is an inversion in the major diastereoisomer, e.g., with 4-methoxybenzaldehyde.

**Table 2:** Prins fluorination microwave (100 W, 50 °C, 10 min) reactions using homoallylic alcohol (**3**), an aldehyde and BF_3_·OEt_2_ in DCM.

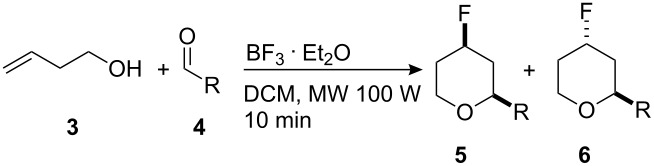			
entry	aldehyde	d.r. (**5**/**6**)	conversion

**5a**	4-nitrobenzaldehyde	1.5/1	53%
**5e**	2-bromobenzaldehyde	3/1	92%
**5f**	3-bromobenzaldehyde	1.8/1	93%
**5g**	4-bromobenzaldehyde	2.3/1	83%
**5i**	4-methoxybenzaldehyde	1/1.2	41%
**5k**	hexanal	1.8/1	91%
**5m**	benzaldehyde	3.4/1	66%

A series of low temperature studies was carried out in an attempt to improve the diastereoselectivity. When the temperature was lowered to −20 °C (Table 3) the diastereoselectivity increased from ~2/1 to 10/1 and generally in good yields, but with a significant increase in the reaction time. Lowering the temperature below −20 °C did not lead to a significant improvement.

**Table 3:** Prins fluorination reaction with alcohol **3** and aldehydes at −20 °C. Reaction conditions: BF_3_·OEt_2_ (1 equiv), but-3-en-1-ol (**3**) (1 equiv), aldehyde (1 equiv), DCM, −20 °C, 5 h.

entry	aldehyde	d.r. (**5/6**)	conversion

**5a**	4-nitrobenzaldehyde	10/1	61%
**5k**	hexanal	10/1	66%
**5m**	benzaldehyde	10/1	59%

**Figure 1:**
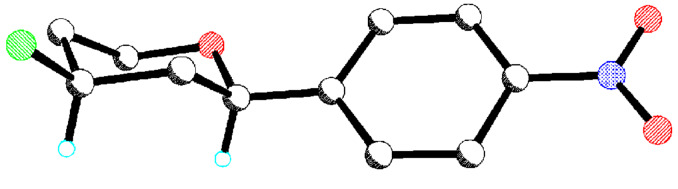
X-ray crystal structure of *syn*-**5a**.

In order to confirm the configuration of the major diastereoisomer, an X-ray structure analysis was carried out on the major diastereoisomer produced in the low temperature reaction between alcohol **3** and 4-nitrobenzaldehyde. The X-ray confirmed that the major diastereoisomer is *syn*-**5a** as shown in Figure 1.

**Scheme 2:**
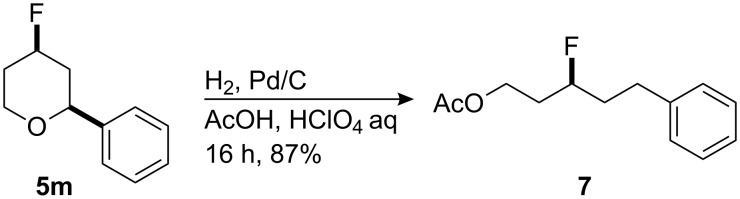
Ring opening hydrogenation of an oxa-Prins product.

In order to elaborate one of the Prins fluorination products, pyran **5m** was subjected to hydrogenolysis [23] as illustrated in Scheme 2. This resulted in the efficient conversion to the corresponding open chain compound 3-fluoro-5-phenylpentyl acetate (**7**).

The structural diversity of the Prins fluorination reaction was extended using 2-vinylcyclohexan-ol (**8**) as a substrate. Vinylcyclohexanol **8** was prepared by treatment of cyclohexene oxide (**10**) with vinylmagnesium bromide in the presence of CuBr/DMSO as illustrated in Table 4 [24]. This gave a single diastereoisomer of **8** which was used in the Prins fluorination reactions.

Prins fluorination reactions, at −20 °C, with cyclohexanol **8** using benzaldehyde and 4-nitrobenzaldehyde gave rise to the corresponding bicyclic products **9** with good diastereoselectivity (10/1) and in moderate yields (Table 4).

**Table 4:** Prins reactions using vinylcyclohexanol **8** and an aldehyde at −20 °C. Reaction conditions: BF_3_·OEt_2_ (1 equiv), 2-vinylcyclohexanol (**8**) (1 equiv), aldehyde (1 equiv), DCM, −20 °C, 5 h.

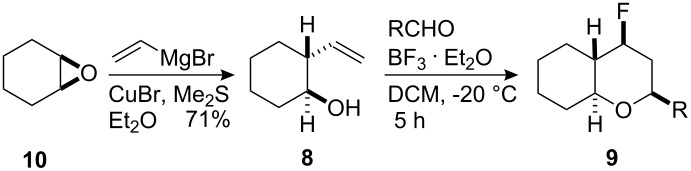			
	aldehyde	*syn/anti*	yield of *syn*-**9**

**9a**	benzaldehyde	10/1	59%
**9b**	4-nitrobenzaldehyde	10/1	57%

**Figure 2:**
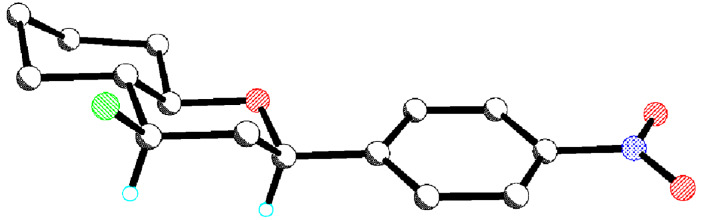
X-ray crystal structure of the major bicyclic tetrahydropyran diastereoisomer **9b**.

The X-ray crystal structure of the predominant bicyclic diastereoisomer was determined and the *syn*-stereoisomer **9b**, as shown in Figure 2, was confirmed as the major product of this Prins reaction.

**Scheme 3:**
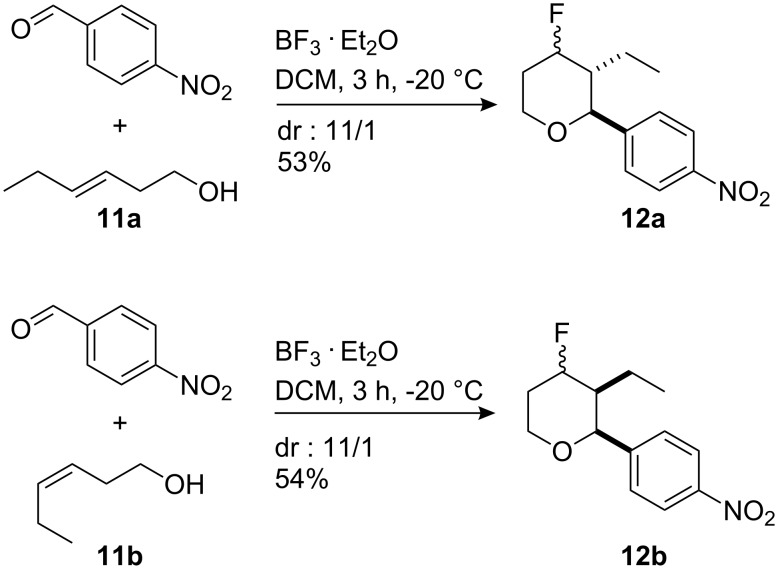
Reaction using (*E*)-**11a** and (*Z*)-**11b** hex-3-en-1-ols with 4-nitrobenzaldehyde to generate 4-fluorotetrahydropyrans **12**.

It is interesting to note that when (*E*)- and (*Z*)-hex-3-en-1-ol (**11a**) and (**11b**) were used as substrates, the double bond stereochemistry is retained. Only two diastereoisomers were observed in the products, differing only in the orientation of the fluorine. In general, these reactions gave good diastereoselectivities in moderate to good yields (Scheme 3).

### Aza-Prins fluorination reaction

The aza-Prins reaction is less well known but has been exploited, e.g., in tandem reactions in alkaloid synthesis [25–26]. Recently, it has been used to incorporate a halogen atom at the 4 position of a piperidine ring as reported by Carballo et al. [16], and most recently an example incorporating fluorine at the 4-position of a piperidine using Et_4_NF·5HF has been reported [14–15]. We have extended this study to explore more fully the aza-Prins fluorination reaction of homoallylamine **13**. Several amine protecting groups such as benzoyl and Boc were examined, but only the *N*-tosyl homoallylamine **13** proved to be a useful substrate as illustrated in Table 5. The tosylamine **13** was prepared (41% yield) by the reaction of 1-bromobut-3-ene with *N*-tosylamine in the presence of potassium carbonate. The yields and diastereoselectivity of the aza-Prins reactions with a variety of aldehydes were comparable to the oxa-Prins reactions, but longer reaction times were required (typically 36 h). The reactions are summarised in Table 5. In contrast to the oxa-Prins reactions, lowering the temperature of the reaction had no measurable influence on the diastereoselectivity (Table 5, entry 4a).

**Table 5:** Aza-Prins reaction between *N*-homoallyl-*N*-tosylamine (**13**) and aldehydes **14** in the presence of BF_3_·OEt_2_. Reaction conditions (except entry 2): Amine **13** (1 equiv), aldehyde (1 equiv), BF_3_·OEt_2_ for 36 h at rt; yield refers to the isolation of both stereoisomers in each case except for entry **14j** and **14k** where the yields refer to the isolation of diastereoisomer mixture.

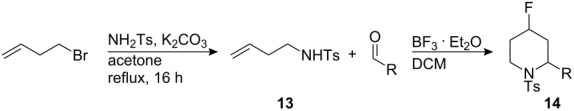			
entry	aldehyde	(*syn/anti* **14**)	conversion

**14a**	4-nitrobenzaldehyde	1/1	65%
**14a**^a^	4-nitrobenzaldehyde	1/1	71%
**14b**	hexanal	2/1	75%
**14c**	4-bromobenzaldehyde	1/1	71%
**14d**	3-bromobenzaldehyde	2/1	61%
**14e**	2-bromobenzaldehyde	1/1.6	50%
**14f**	4-fluorobenzaldehyde	1.8/1	70%
**14g**	3-fluorobenzaldehyde	2/1	67%
**14h**	4-methoxybenzaldehyde	2.5/1	23%
**14i**	2-methylcinnamaldehyde	no reaction	–
**14j**	acetaldehyde	1/1	73%
**14k**	isobutyraldehyde	1/1	82%

^a^Low temperature reaction (−20 °C, 48 h)

All the aldehydes used in the study (Table 5) gave universally poor diastereoselectivities, however the conversions were generally good except in the case of where a relatively strong electron donating group was present on the aromatic ring (e.g. **14h**, Table 5). There was no reaction with the α,β-unsaturated aldehyde, 2-methylcinnamaldehyde (**14i**). In the case of aliphatic aldehydes (**14b**, **14j**, **14k**), the conversions were high, but again the diastereoselectivity was poor.

**Microwave – aza-Prins:** Following the observation of reduced reaction times in the oxa-Prins under microwave conditions, it was of interest to investigate the aza-Prins fluorination reaction under microwave conditions, particularly as these reactions were much slower.

In the event, the microwave reactions proved to be very efficient giving products with improved conversions after 30 min as shown in Table 6. Despite this improvement in rate, there was no significant improvement in the diastereoselectivity of the reaction products. This should be contrasted with the study using an HF containing ionic liquid (Et_4_NF·5HF) in place of BF_3_·OEt_2_, where diastereoselectivities for similar aza-Prins reactions where around 7:1 to 10:1 in favour of the *syn*-products [14–15].

**Table 6:** aza-Prins reaction under microwave conditions. Reaction conditions: amine **13** (1 equiv), aldehyde (1 equiv), BF_3_·OEt_2_, microwave 100 W, 50 °C, 30 min.

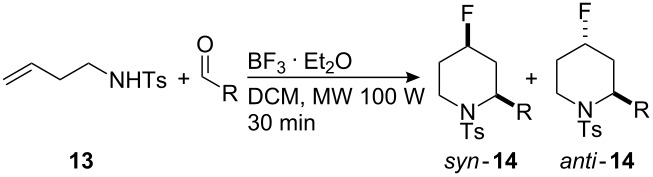			
entry	aldehyde	*syn/anti*	conversion

**14a**	4-nitrobenzaldehyde	1.3/1	61%
**14b**	hexanal	1.9/1	83%
**14c**	4-bromobenzaldehyde	1.9/1	68%
**14f**	4-fluorobenzaldehyde	1.5/1	63%
**14i**	acetaldehyde	1.2/1	77%
**14k**	isobutyraldehyde	1.9/1	83%

**Figure 3:**
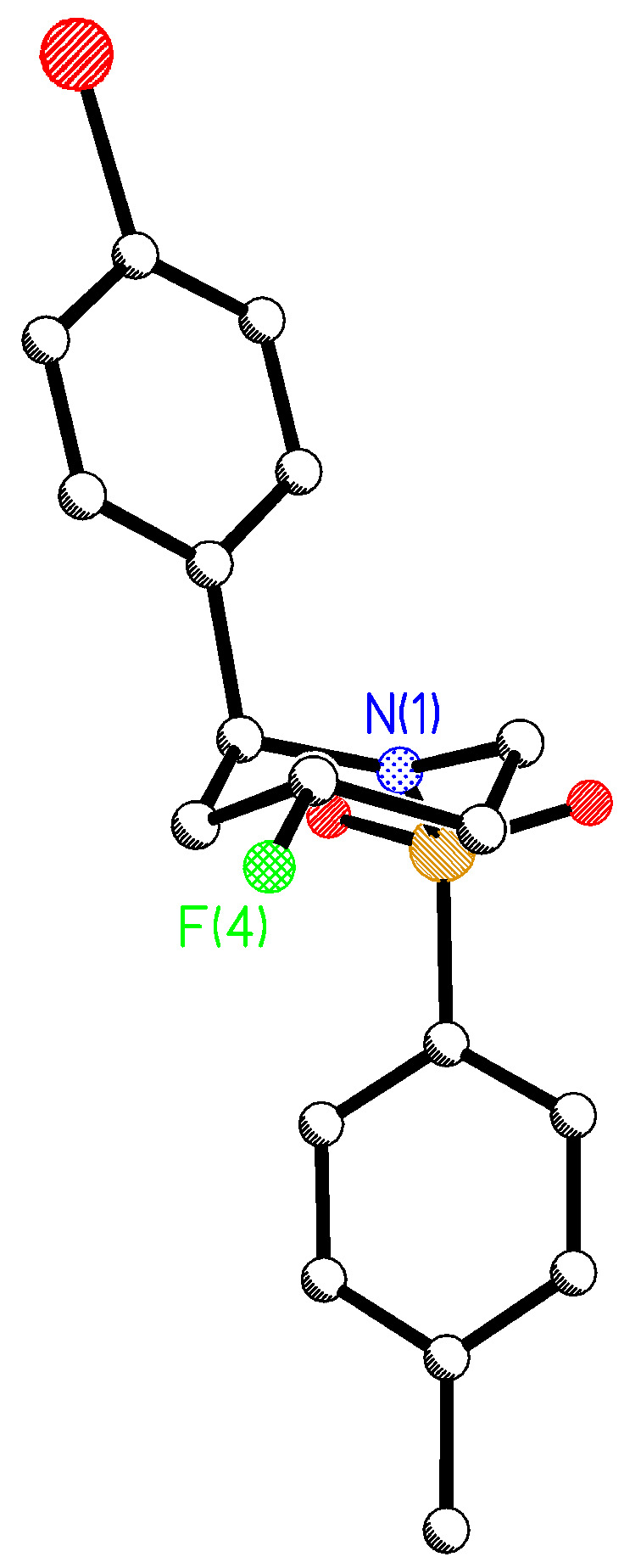
X-ray crystal structure of the minor *anti*-piperidine product **14d**.

The crystal structure of the minor *anti*-diastereoisomer of the piperidine product **14d** was determined and is shown in Figure 3.

## Conclusion

Selective methods for fluorination are finding increasing utility in pharmaceutical, agrochemicals and fine chemicals research. The BF_3_·OEt mediated oxa- and aza-Prins fluorination extends the methodologies available for the synthesis of C–F bonds, particularly concomitant with *O*- and *N*-heterocycle assembly. In general the diastereoselectivities are poor in these reactions, however they can be improved in the oxa-Prins case by lowering the temperature of the reactions to −20 °C. Both these oxa- and aza-Prins reactions can be significantly accelerated under microwave conditions.

## Supporting Information

File 1 Experimental and characterisation details of synthesised compounds.
