# Supplementary material for: Prins fluorination cyclisations: Preparation of 4-fluoro-pyran and -piperidine heterocycles
Source: Beilstein J Org Chem. 2010 Apr 26;6:41. doi: 10.3762/bjoc.6.41 (PMC2874316; doi:10.3762/bjoc.6.41)
Supplement: File 1 — Experimental and characterisation details of synthesised compounds. [file Beilstein_J_Org_Chem-06-41-s001.pdf]

Supporting Information to

The Prins fluorination cyclisations: Preparation of 4-fluoro-pyran and piperidine heterocycles.

Guillaume G. Launay, Alexandra M. Z. Slawin and David O'Hagan*

Address: School of Chemistry and Centre for Biomolecular Sciences , University of St Andrews, North Haugh, St Andrews, Fife, KY16 9ST, United Kingdom.

Email: David O'Hagan - do1@st-andrews.ac.uk

* Corresponding author

Experimental and characterisation details of synthesised compounds

General method for the oxa-Prins reaction

Boron trifluoride diethyl etherate (0.5 mmol, 1.0 equiv) was added to a solution of the aldehyde (0.5 mmol, 1 equiv) in dichloromethane. After 5 min, the alcohol (0.5 mmol, 1 equiv) was added, and the mixture stirred for 5 h. Water and dichloromethane were then added and the layers were separated. The aqueous layer was extracted with dichloromethane, the organic layers dried, filtered and concentrated. The residue was then purified on silica.

General method for the aza-Prins reaction

Boron trifluoride diethyl etherate (0.5 mmol, 1.0 equiv) was added to a solution of the aldehyde (0.5 mmol, 1 equiv) in dichloromethane. After 5 min, the *N*-(tosyl)-*N*-but-3-ene (0.5 mmol, 1 equiv) was added, and the mixture stirred 36 h. Water and dichloromethane were then added and the layers were separated. The aqueous layer was extracted with dichloromethane, the organic layers dried, filtered and concentrated. The residue was then purified on silica.

General method for the oxa-Prins reaction under microwave conditions

Boron trifluoride diethyl etherate (0.5 mmol, 1.0 equiv) was added to a solution of the aldehyde (0.5 mmol, 1 equiv) in dichloromethane. After 5 min, the alcohol (0.5 mmol, 1 equiv) was added, and the mixture irradiated for 10 min with microwaves (100 W). Water and dichloromethane were then added and the layers were separated. The aqueous layer was extracted with dichloromethane, the organic layers dried, filtered and concentrated. The residue was then purified on silica.

General method for the aza-Prins reaction under microwave conditions

Boron trifluoride diethyl etherate (0.5 mmol, 1.0 equiv) was added to a solution of the aldehyde (0.5 mmol, 1 equiv) in dichloromethane. After 5 min, the *N*-(tosyl)-*N*-but-3-ene (0.5 mmol, 1 equiv) was added, and the mixture irradiated for 30 min by microwaves (100 W). Water and dichloromethane were then added and the layers were separated. The aqueous layer was extracted with dichloromethane, the organic layers dried, filtered and concentrated. The residue was then purified on silica.

2-(4-Nitrophenyl)-4-fluoropyrans *syn*-5a and *anti*-5a.

Products ***syn*-5a and *anti*-5a** were prepared according to the general procedure outlined for the oxa-Prins reaction from 4-nitrobenzaldehyde (76 mg, 0.5 mmol), but-3-en-1-ol **3** (45 µL, 0.5 mmol) and boron trifluoride (65 µL, 0.5 mmol).

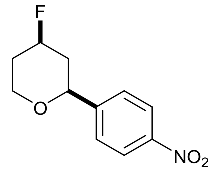

***syn*-5a**

Product ***syn*-5a** was isolated after purification on silica gel (hexane/diethyl ether, 8/2) as a white solid (47 mg, 41%, microwave: 33 mg, 29%, at -20 °C: 70mg, 61%).
NMR: ^{1}H, 300 MHz (δ in ppm): 8.24-8.14 (2H, m, ar), 7.56-7.47 (2H, m, ar), 4.84 (1H, dtt, *J*= 49.0 Hz, 10.9 Hz, 4.9 Hz, C*H*F), 4.43 (1H, dt, *J*= 11.5 Hz, 1.8 Hz, OC*H*CH_2), 4.25 (1H, dtd, J= 11.9 Hz, 5.7 Hz, 1.8 Hz, OC*H*HCH_2), 3.58 (1H, tt, *J*= 12.3 Hz, 1.8 Hz, OC*H*HCH_2), 2.39 (1H, dtt, *J*= 12.3 Hz, 4.9 Hz, 2.1 Hz, OCHC*H*H), 2.20-2.09 (1H, m, OCH_2C*H*H), 1.86 (1H, tddd, J= 12.3 Hz, 11.1 Hz, 9.9 Hz, 5.3 Hz, OCH_2C*H*H), 1.67 (1H, dtd, J= 12.3 Hz, 11.5 Hz, 9.5, OCHC*H*H); **^{13}C, 75 MHz (δ in ppm):** 148.6 (Cq, ar), 147.4 (Cq, ar), 126.4 (CH, ar), 123.6 (CH, ar), 88.7 (CH, d, *J*= 177.7 Hz, *C*HF), 76.4 (CH, d, *J*= 11.5 Hz, O*C*HCH_2), 65.3 (CH_2, d, *J*= 11.6 Hz, O*C*H_2CH_2), 40.4 (CH_2, d, *J*= 17.7 Hz, OCH*C*H_2), 32.6 (CH_2, d, *J*= 17.7 Hz, OCH_2*C*H_2); **^{19}F, 282 MHz (δ in ppm):** -170.5 to -170.8 (m); **HRMS m/z:** $[MH]^+$: 226.0879, calculated 226.0878; **Mp:** 80-82 °C; **IR: ν_{max} (neat)/cm^{-1}:** 2976, 2942, 2853, 1521, 1348, 1320, 1181, 1133, 1078, 849.

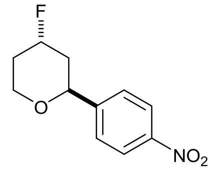

***anti*-5a**

Product ***anti*-5a** was isolated after purification on silica gel (hexane/diethyl ether, 8/2) as a white solid (23 mg, 20%, microwave: 20 mg, 18%).

NMR: ^{1}H, 300 MHz (δ in ppm): 8.26-8.18 (2H, m, ar), 7.57-7.50 (2H, m, ar), 5.11 (1H, dtt, *J*= 47.7 Hz, 2.9 Hz, 2.6 Hz, C*H*F), 4.87 (1H, dd, *J*= 11.8 Hz, 2.2 Hz, OC*H*CH_2), 4.10-3.97 (2H, m, OC$H_2$$CH_2$), 2.21 (1H, dddt, *J*= 14.4 Hz, 10.7 Hz, 3.3 Hz, 2.2 Hz, OCHC*H*H), 2.06-1.87 (2H, m, OCH_2CH_2), 1.74 (1H, dddd, *J*= 43.0Hz, 14.1Hz, 5.9Hz, 2.2Hz, OCHC*H*H); **^{13}C, 75 MHz (δ in ppm):** 149.6 (Cq, ar), 147.3 (Cq, ar), 126.3 (CH, ar), 123.6 (CH, ar), 86.3 (CH, d, *J*= 169.6 Hz, *C*HF), 73.1 (CH, O*C*HCH_2), 63.0 (CH_2, d, *J*= 11.6 Hz, O*C*$H_2$$CH_2$), 38.7 ($CH_2$, d, *J*= 20.3 Hz, OCH*C*H_2), 30.4 (CH_2, d, *J*= 21.1 Hz, OCH_2*C*H_2); **^{19}F, 282 MHz (δ in ppm):** -185.7 to -186.3 (m); **HRMS m/z:** $[MH]^+$: 226.0874, calculated 226.0879; **Mp :** 100-102 °C; **IR: ν_{max} (neat)/cm^{-1}:** 3080, 2974, 2852, 1514, 1345, 1207, 1150, 1069, 872, 857.

2-(2-Fluorophenyl)-4-fluoropyrans *syn*-5b and *anti*-5b.

Products ***syn*-5b and *anti*-5b** were prepared according to the general procedure outlined for the oxa-Prins reaction from 2-fluorobenzaldehyde (53 µL, 0.5 mmol), but-3-en-1-ol **3** (45 µL, 0.5 mmol) and boron trifluoride (65 µL, 0.5 mmol).

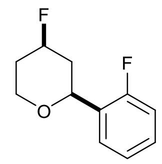

***syn*-5b**

Product ***syn*-5b** was isolated after purification on silica gel (hexane/diethyl ether, 8/2) as a colorless oil (50 mg, 50%).

NMR: ^{1}H, 300 MHz (δ in ppm): 7.50 (1H, td, *J*= 7.5 Hz, 5.6 Hz, ar), 7.32-7.23 (1H, m, ar), 7.17 (1H, td, *J*= 7.5 Hz, 1.2 Hz, ar), 7.03 (1H, ddd, *J*= 10.6 Hz, 8.2 Hz, 1.2 Hz, ar), 4.84 (1H, dtt, *J*= 49.2 Hz, 11.0 Hz, 5.0 Hz, C*H*F), 4.66 (1H, dt, *J*= 11.4 Hz, 1.7 Hz, OC*H*CH_2), 4.23 (1H, dtd, *J*= 12.0 Hz, 5.7 Hz, 1.7 Hz, OC*H*HCH_2), 3.60 (1H, tt, *J*= 12.3 Hz, 1.9 Hz, OC*H*HCH_2), 2.38 (1H, dtt, *J*= 12.3 Hz, 5.0 Hz, 1.8 Hz, OCHC*H*H), 2.20-2.09 (1H, m, OCH_2C*H*H), 1.87 (1H, tddd, *J*= 12.5 Hz, 11.0 Hz, 10.1 Hz, 5.2 Hz,

OCH_2CHH), 1.72 (1H, dtd, *J*= 12.1 Hz, 11.0 Hz, 9.5 Hz, OCHC*H*H); **^{13}C, 75 MHz (δ in ppm):** 159.2 (Cq, d, *J* = 246.0 Hz, *C*ar-F), 129.1 (CH, d, *J*= 8.0 Hz, ar), 128.4 (Cq, ar), 127.1 (CH, d, *J*= 4.2 Hz, ar), 124.4 (CH, d, *J*= 3.5 Hz, ar), 115.2 (CH, d, *J*= 21.7 Hz, ar), 89.0 (CH, d, *J*= 176.9 Hz, *C*HF), 71.4 (CH, dd, *J*= 12.6 Hz, 3.6 Hz, $OCHCH_2$), 65.5 (CH_2, d, *J*= 11.7 Hz, OCH_2CH_2), 39.5 (CH_2, d, *J*= 18.1 Hz, $OCHCH_2$), 32.9 (CH_2, d, *J*= 18.1 Hz, OCH_2CH_2); **^{19}F, 282 MHz (δ in ppm):** -120.3 to -120.4 (m), -170.3- -170.7 (m); **HRMS m/z:** [MH, -HF]$^+$: 179.0871, calculated 179.0872; **IR: v_{max} (neat)/cm^{-1}:** 3047, 2961, 2932, 2855, 1589, 1494, 1455, 1230, 1183, 1159, 1084, 1044, 982.

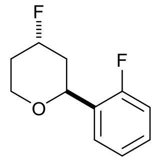

***anti*-5b**

Product ***anti*-5b** was isolated after purification on silica gel (hexane/diethylether, 8/2) as a colorless oil (10 mg, 10%).

NMR: ^{1}H, 300 MHz (δ in ppm): 7.48 (1H, td, *J*= 7.5 Hz, 1.9 Hz, ar), 7.30-7.21 (1H, m, ar), 7.15 (1H, td, *J*= 7.5 Hz, 1.4 Hz, ar), 7.03 (1H, ddd, *J*= 10.5 Hz, 8.1 Hz, 1.4 Hz, ar), 5.09 (1H, dquint, *J*= 48.1 Hz, 2.9 Hz, C*H*F), 5.08 (1H, dd, *J*= 11.7 Hz, 2.0 Hz, $OCHCH_2$), 4.07-4.00 (2H, m, OCH_2CH_2), 2.28-2.16 (1H, m, OCHC*H*H), 2.07-1.69 (3H, m, OCH_2CH_2, OCHC*H*H); **^{13}C, 75 MHz (δ in ppm):** 159.2 (Cq, d, *J* = 246.0 Hz, *C*ar-F), 129.2 (CH, d, *J*= 8.1 Hz, ar), 126.9 (CH, d, *J*= 4.0 Hz, ar), 126.4 (Cq, ar), 124.4 (CH, d, *J*= 3.5 Hz, ar), 115.3 (CH, d, *J*= 21.3 Hz, ar), 86.8 (CH, d, *J*= 169.1 Hz, *C*HF), 68.9 (CH, $OCHCH_2$), 63.3 (CH_2, OCH_2CH_2), 37.7 (CH_2, d, *J*= 22.3 Hz, $OCHCH_2$), 30.3 (CH_2, d, *J*= 22.4 Hz, OCH_2CH_2); **^{19}F, 282 MHz (δ in ppm):** -119.5 to -119.6 (m), -186.4 to -187.0 (m); **HRMS m/z:** [MH, -HF]$^+$: 179.0874, calculated 179.0872; **IR: v_{max} (neat)/cm^{-1}:** 3017, 2918, 2852, 1589, 1491, 1460, 1270, 1183, 1135, 1089, 962.

2-(3-Fluorophenyl)-4-fluoropyrans *syn*-5c and *anti*-5c.

Products ***syn*-5c** and ***anti*-5c** were prepared according to the general procedure outlined for the oxa-Prins reaction from 3-fluorobenzaldehyde (55 µL, 0.5 mmol), but-3-en-1-ol **3** (45 µL, 0.5 mmol) and boron trifluoride (65 µL, 0.5 mmol).

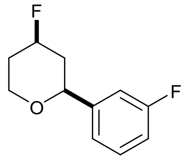

***syn*-5c**

Product ***syn*-5c** was isolated after purification on silica gel (hexane/diethyl ether, 8/2) as a colorless oil (55 mg, 55%).

NMR: ^{1}H, 300 MHz (δ in ppm): 7.31-7.19 (1H, m, ar), 7.09-7.00 (2H, m, ar), 6.93 (1H, tdd, *J*= 8.4 Hz, 2.6 Hz, 1.1 Hz, ar), 4.76 (1H, dtt, *J*= 49.1 Hz, 11.0 Hz, 5.0 Hz, C*H*F), 4.27 (1H, dt, *J*= 11.5 Hz, 1.8 Hz, OC*H*CH_2), 4.16 (1H, dtd, *J*= 12.0 Hz, 5.7 Hz, 1.8 Hz, OC*H*HCH_2), 3.50 (1H, tt, *J*= 12.3 Hz, 1.8 Hz, OC*H*HCH_2), 2.29 (1H, dtt, *J*= 12.3 Hz, 4.9 Hz, 2.1 Hz, OCHC*H*H), 2.13-2.01 (1H, m, OCH_2C*H*H), 1.79 (1H, tddd, *J*= 12.5 Hz, 11.0 Hz, 9.9 Hz, 5.0 Hz, OCH_2C*H*H), 1.65 (1H, dtd, *J*= 12.2 Hz, 11.3 Hz, 9.6 Hz, OCHC*H*H); **^{13}C, 75 MHz (δ in ppm):** 162.8 (Cq, d, *J* = 247.0 Hz, *C*ar-F), 143.9 (Cq, dd, *J*= 8.6 Hz, 1.2 Hz, ar), 129.9 (CH, d, *J*= 8.2 Hz, ar), 121.3 (CH, d, *J*= 2.8 Hz, ar), 114.6 (CH, d, *J*= 21.9 Hz, ar), 112.8 (CH, d, *J*= 21.9 Hz, ar), 89.2 (CH, d, *J*= 176.2 Hz, *C*HF), 77.0 (CH, d, *J*= 12.7 Hz, O*C*HCH_2), 65.4 (CH_2, d, *J*= 11.5 Hz, O*C*H_2CH_2), 40.5 (CH_2, d, *J*= 17.3 Hz, OCH*C*H_2), 32.9 (CH_2, d, *J*= 17.3 Hz, OCH_2*C*H_2); **^{19}F, 282 MHz (δ in ppm):** -113.3 to -113.4 (m), -170.3 to -170.7 (m); **HRMS m/z:** [MH, -HF]$^{+}$: 179.0870, calculated 179.0872; **IR: v_{max} (neat)/cm^{-1}:** 3017, 2919, 2854, 1590, 1491, 1448, 1272, 1186, 1138, 1073, 962.

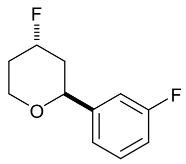

***anti*-5c**

Product ***anti*-5c** was isolated after purification on silica gel (hexane/diethyl ether, 8/2) as a colorless oil (7 mg, 7%).

NMR: ^{1}H, 300 MHz (δ in ppm): 7.35-7.28 (1H, m, ar), 7.16-7.07 (2H, m, ar), 7.0-6.93 (1H, m, ar), 5.10 (1H, dquint, *J*= 47.8 Hz, 2.8 Hz, C*H*F), 4.76 (1H, dd, *J*= 12.2 Hz, 2.5 Hz, OC*H*CH$_2$), 4.05-3.99 (2H, m, OC*H*$_2$CH$_2$), 2.23-2.13 (1H, m, OCHC*H*H), 2.06-1.88 (2H, m, OCH$_2$C*H*$_2$), 1.79 (1H, dddd, *J*= 43.5 Hz, 14.5 Hz, 11.5 Hz, 2.1 Hz, OCHC*H*H) ; **^{13}C, 75 MHz (δ in ppm):** 162.9 (Cq, *J* = 249.3 Hz, *C*ar-F), 147.1 (Cq, ar), 129.9 (CH, d, *J*= 9.7 Hz, ar), 121.2 (CH, d, *J*= 2.8 Hz, ar), 114.4 (CH, d, *J*= 22.5 Hz, ar), 112.7 (CH, d, *J*= 22.5 Hz, ar), 86.7 (CH, d, *J*= 169.1 Hz, *C*HF), 73.4 (CH, O*C*HCH$_2$), 63.1 (CH$_2$, O*C*H$_2$CH$_2$), 38.6 (CH$_2$, d, *J*= 21.9 Hz, OCH*C*H$_2$), 30.5 (CH$_2$, d, *J*= 21.9 Hz, OCH$_2$*C*H$_2$) ; **^{19}F, 282 MHz (δ in ppm):** -113.4 to -113.5 (m), -186.1 to -186.5 (m) ; **HRMS m/z:** [MH, -HF]$^{+}$: 179.0882, calculated 179.0872; **IR: ν_{max} (neat)/cm^{-1}:** 3075, 2962, 2856, 1592, 1488, 1447, 1257, 1174, 1139, 1082, 984.

2-(4-Fluorophenyl)-4-fluoropyrans *syn*-5d and *anti*-5d.

Products ***syn*-5d** and ***anti*-5d** were prepared according to the general procedure outlined for the oxa-Prins reaction from 4-fluorobenzaldehyde (53 µL, 0.5 mmol), but-3-en-1-ol **3** (45 µL, 0.5 mmol) and boron trifluoride (65 µL, 0.5 mmol).

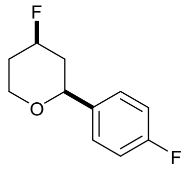

***syn*-5d**

Product ***syn*-5d** was isolated after purification on silica gel (hexane/diethyl ether, 8/2) as a colorless oil (49 mg, 49%).

NMR: ^{1}H, 400 MHz (δ in ppm): 7.37-7.30 (2H, m, ar), 7.08-7.01 (2H, m, ar), 4.82 (1H, dtt, *J*= 49.1 Hz, 11.0 Hz, 5.9 Hz, C*H*F), 4.31 (1H, dt, *J*= 11.6 Hz, 1.8 Hz, OC*H*CH$_2$), 4.21 (1H, dtd, *J*= 11.9 Hz, 5.7 Hz, 1.7 Hz, OC*H*HCH$_2$), 3.57 (1H, tt, *J*= 12.3 Hz, 1.8 Hz, OC*H*HCH$_2$), 2.32 (1H, dtt, *J*= 12.3 Hz, 4.8 Hz, 2.1 Hz, OCHC*H*H), 2.17-2.09 (1H, m, OCH$_2$C*H*H), 1.85 (1H, tddd, *J*= 12.5 Hz, 11.0 Hz, 9.9 Hz, 5.1 Hz, OCH$_2$C*H*H), 1.74 (1H, dtd, *J*= 12.2 Hz, 11.3 Hz, 9.6 Hz, OCHC*H*H); **^{13}C, 100 MHz (δ in ppm):** 162.3 (CH, d, *J*= 249.0 Hz, ar), 137.9 (Cq, ar), 127.6 (CH, d, *J*= 8.5 Hz, ar),

115.3 (CH, d, *J*= 21.2 Hz, ar), 89.2 (CH, d, *J*= 177.2 Hz, *C*HF), 77.2 (CH, d, *J*= 11.3 Hz, O*C*HCH$_2$), 65.4 (CH$_2$, d, *J*= 11.8 Hz, O*C*H$_2$CH$_2$), 40.5 (CH$_2$, d, *J*= 17.6 Hz, OCH*C*H$_2$), 32.9 (CH$_2$, d, *J*= 17.7 Hz, OCH$_2$*C*H$_2$); **^{19}F, 376 MHz (δ in ppm):** -115.0 to -115.1 (m), -170.3 to -170.6 (m); **HRMS m/z:** [MH, -HF]$^+$: 179.0870, calculated 179.0872; **IR: ν_{max} (neat)/cm^{-1}:** 2972, 2929, 1498, 1447, 1290, 1186, 1137, 1080, 834.

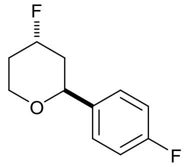

***anti*-5d**

Product ***anti*-5d** was isolated after purification on silica gel (hexane/diethyl ether, 8/2) as a colorless oil (10 mg, 10%).

NMR: ^{1}H, 400 MHz (δ in ppm): 7.36-7.30 (2H, m, ar), 7.08-7.00 (2H, m, ar), 5.10 (1H, dquint, *J*= 48.0 Hz, 2.6 Hz, C*H*F), 4.74 (1H, dd, *J*= 12.0 Hz, 2.3 Hz, OC*H*CH$_2$), 4.04-3.98 (2H, m, OC*H*$_2$CH$_2$), 2.20-2.10 (1H, m, OCHC*H*H), 2.06-1.8 (2H, m, OCH$_2$C*H*$_2$), 1.81 (1H, dddd, *J*= 43.7 Hz, 14.4 Hz, 11.8 Hz, 2.2 Hz, OCHC*H*H); **^{13}C, 100 MHz (δ in ppm):** 162.1 (Cq, d, *J*= 245.0 Hz), 137.9 (Cq, ar), 127.4 (CH, d, *J*= 8.1 Hz, ar), 115.2 (CH, d, *J*= 20.5 Hz, ar), 86.7 (CH, d, *J*= 168.1 Hz, *C*HF), 73.4 (CH, O*C*HCH$_2$), 63.1 (CH$_2$, O*C*H$_2$CH$_2$), 38.6 (CH$_2$, d, *J*= 20.7 Hz, OCH*C*H$_2$), 30.5 (CH$_2$, d, *J*= 20.5 Hz, OCH$_2$*C*H$_2$); **^{19}F, 376 MHz (δ in ppm):** -115.4 to -115.5 (m), -186.1 to -186.5 (m); **HRMS m/z :** [MH, -HF]$^+$: 179.0874, calculated 179.0872; **IR: ν_{max} (neat)/cm^{-1}:** 2977, 2926, 1447, 1276, 1181, 1123, 1086, 977.

2-(2-Bromophenyl)-4-fluoropyrans *syn*-5e and *anti*-5e.

Products ***syn*-5e** and ***anti*-5e** were prepared according to the general procedure outlined for the oxa-Prins reaction from 2-bromobenzaldehyde (58 µL, 0.5 mmol), but-3-en-1-ol **3** (45 µL, 0.5 mmol) and boron trifluoride (65 µL, 0.5 mmol).

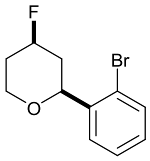

***syn*-5e**

Product ***syn*-5e** was isolated after purification on silica gel (hexane/diethyl ether, 8/2) as a colorless oil (68 mg, 52%, microwave: 87 mg, 67%).

NMR: ^{1}H, 300 MHz (δ in ppm): 7.59-7.50 (2H, m, ar), 7.40-7.32 (1H, td, *J*= 7.5 Hz, 1.1 Hz, ar), 7.20-7.12 (1H, td, *J*= 7.8 Hz, 1.7 Hz, ar), 4.83 (1H, dtt, *J*= 49.0 Hz, 10.1 Hz, 5.0 Hz, C*H*F), 4.65 (1H, dt, *J*= 11.3 Hz, 1.9 Hz, OC*H*CH_2), 4.23 (1H, dtd, *J*= 11.7 Hz, 5.7 Hz, 1.6 Hz, OC*H*HCH_2), 3.61 (1H, tt, *J*= 12.3 Hz, 1.8 Hz, OC*H*HCH_2), 2.52 (1H, dtt, *J*= 12.3 Hz, 4.8 Hz, 2.1 Hz, OCHC*H*H), 2.20-2.09 (1H, m, OCH_2C*H*H), 1.87 (1H, tddd, *J*= 12.5 Hz, 10.8 Hz, 10.0 Hz, 5.1 Hz, OCH_2C*H*H), 1.54 (1H, dtd, *J*= 12.3 Hz, 11.3 Hz, 9.3 Hz, OCHC*H*H); **^{13}C, 75 MHz (δ in ppm):** 140.5 (Cq, ar), 132.5 (CH, ar), 129.0 (CH, ar), 127.8 (CH, ar), 127.4 (CH, ar), 121.3 (Cq, ar), 88.9 (CH, d, *J*= 177.2 Hz, *C*HF), 76.7 (CH, d, *J*= 12.2 Hz, O*C*HCH_2), 65.4 (CH_2, d, *J*= 11.9 Hz, O*C*H_2CH_2), 39.2 (CH_2, d, *J*= 17.4 Hz, OCH*C*H_2), 32.9 (CH_2, d, *J*= 17.7 Hz, OCH_2*C*H_2); **^{19}F, 376 MHz (δ in ppm):** -170.1 to -170.5 (m); **HRMS m/z:** [MH, -HF]$^+$: 239.0072; 241.0061, calculated 239.0072, 241.0051; **IR: ν_{max} (neat)/cm^{-1}:** 3067, 2962, 2854, 1568, 1473, 1440, 1371, 1249, 1158, 1082, 1082, 982, 754, 679.

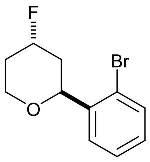

***anti*-5e**

Product ***anti*-5e** was isolated after purification on silica gel (hexane/diethyl ether, 8/2) as a colorless oil (10 mg, 8%, microwave: 26 mg, 20%).

NMR: ^{1}H, 300 MHz (δ in ppm): 7.57-7.50 (2H, m, ar), 7.38-7.30 (1H, m, ar), 7.17-7.10 (1H, m, ar), 5.10 (1H, dd, *J*= 11.6 Hz, 2.1 Hz, OC*H*CH_2), 5.09 (1H, dquint, *J*= 47.8 Hz, 2.8 Hz, C*H*F), 4.08-4.03 (2H, m, OC*H*$_2CH_2$), 2.45-2.33 (1H, m, OCHC*H*H), 2.12-1.81 (2H, m, OCH_2C*H*$_2$), 1.60 (1H, dddd, *J*= 43.8 Hz, 14.4 Hz, 11.55 Hz, 2.1

Hz, OCHC*H*H); **^{13}C, 75 MHz (δ in ppm):** 141.5 (Cq, ar), 132.6 (CH, ar), 128.8 (CH, ar), 127.7 (CH, ar), 127.2 (CH, ar), 121.5 (Cq, ar), 86.5 (CH, d, *J*= 168.9 Hz, *C*HF), 73.3 (CH, O*C*HCH$_2$), 63.2 (CH$_2$, O*C*H$_2$CH$_2$), 37.2 (CH$_2$, d, *J*= 20.6 Hz, OCH*C*H$_2$), 30.6 (CH$_2$, d, *J*= 19.9 Hz, OCH$_2$*C*H$_2$); **^{19}F, 282 MHz (δ in ppm):** -186.8 to -187.4 (m); **HRMS m/z:** [MH, -HF]$^+$: 239.0076; 241.0051, calculated 239.0072, 241.0051; **IR: ν_{max} (neat)/cm^{-1}:** 3064, 2953, 2864, 1567, 1470, 1428, 1369, 1254, 1147, 1072, 1021, 982, 751, 705.

2-(3-Bromophenyl)-4-fluoropyrans *syn*-5f and *anti*-5f.

Products ***syn*-5f** and ***anti*-5f** were prepared according to the general procedure outlined for the oxa-Prins reaction from 3-bromobenzaldehyde (58 µL, 0.5 mmol), but-3-en-1-ol **3** (45 µL, 0.5 mmol) and boron trifluoride (65 µL, 0.5 mmol).

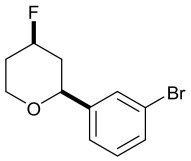

***syn*-5f**

Product ***syn*-5f** was isolated after purification on silica gel (hexane/diethyl ether, 8/2) as a colorless oil (71 mg, 55%, microwave: 72 mg, 55%).

NMR: ^{1}H, 300 MHz (δ in ppm): 7.56-7.53 (1H, m, ar), 7.44 (1H, dt, *J*= 7.3 Hz, 1.9 Hz), 7.30-7.20 (2H, m, ar), 4.82 (1H, dtt, *J*= 48.9 Hz, 11.0 Hz, 5.0 Hz, C*H*F), 4.30 (1H, dt, *J*= 11.7 Hz, 2.0 Hz, OC*H*CH$_2$), 4.22 (1H, dtd, *J*= 12.2 Hz, 5.6 Hz, 1.8 Hz, OC*H*HCH$_2$), 3.56 (1H, tt, *J*= 12.2 Hz, 1.8 Hz, OC*H*HCH$_2$), 2.34 (1H, dtt, *J*= 12.3 Hz, 5.0 Hz, 2.0 Hz, OCHC*H*H), 2.19-2.08 (1H, m, OCH$_2$C*H*H), 1.86 (1H, tddd, *J*= 12.3Hz, 11.0 Hz, 9.8 Hz, 5.0 Hz, OCH$_2$C*H*H), 1.71 (1H, dtd, *J*= 11.9 Hz, 11.5 Hz, 9.6 Hz, OCHC*H*H); **^{13}C, 75 MHz (δ in ppm):** 143.5 (Cq, ar), 130.8 (CH, ar), 130.0 (CH, ar), 128.9 (CH, ar), 124.3 (CH, ar), 122.5 (Cq, ar), 89.0 (CH, d, *J*= 177.0 Hz, *C*HF), 76.9 (CH, d, *J*= 12.4 Hz, O*C*HCH$_2$), 65.4 (CH$_2$, d, *J*= 12.4 Hz, O*C*H$_2$CH$_2$), 40.5 (CH$_2$, d, *J*= 17.8 Hz, OCH*C*H$_2$), 32.8 (CH$_2$, d, *J*= 17.8 Hz, OCH$_2$*C*H$_2$); **^{19}F, 282 MHz (δ in ppm):** -170.3 to -170.7 (m); **HRMS m/z:** [MH, -HF]$^+$: 239.0102; 241.0059, calculated

239.0072, 241.0051; **IR: ν_{max} (neat)/cm^{-1}:** 3066, 2960, 2853, 1568, 1474, 1428, 1369, 1249, 1158, 1082, 1041, 783, 695, 681.

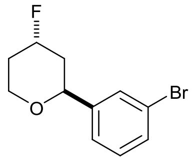

***anti*-5f**

Product ***anti*-5f** was isolated after purification on silica gel (hexane/diethyl ether, 8/2) as a colorless oil (15 mg, 11%, microwave: 34 mg, 26%).

NMR: ^{1}H, 300 MHz (δ in ppm): 7.56-7.51 (1H, m, ar), 7.41 (1H, dt, *J*= 7.4 Hz, 1.8 Hz, ar), 7.29-7.18 (2H, m, ar), 5.09 (1H, dquint, *J*= 47.9 Hz, 2.8Hz, C*H*F), 4.73 (1H, dd, *J*= 11.7 Hz, 2.2 Hz, OC*H*CH_2), 4.05-3.97 (2H, m, OC$H_2$$CH_2$), 2.24-2.10 (1H, m, OCHC*H*H), 2.09-1.82 (2H, m, OCH_2CH_2), 1.78 (1H, dddd, *J*= 43.4 Hz, 14.5 Hz, 11.7 Hz, 2.1 Hz OCHC*H*H); **^{13}C, 75 MHz (δ in ppm):** 144.5 (Cq, ar), 130.6 (CH, ar), 130.0 (CH, ar), 128.9 (CH, ar), 124.3 (CH, ar), 122.6 (Cq, ar), 86.6 (CH, d, *J*= 169.0 Hz, *C*HF), 73.3 (CH, O*C*HCH_2), 63.0 (CH_2, O*C*$H_2$$CH_2$), 38.6 ($CH_2$, d, *J*= 21.1 Hz, OCH*C*H_2), 30.5 (CH_2, d, *J*= 20.0 Hz, OCH_2*C*H_2); **^{19}F, 282 MHz (δ in ppm):** -186.0 to -186.7 (m); **HRMS m/z:** $[MH, -HF]^+$: 239.0076; 241.0051, calculated 239.0072, 241.0051; **IR: ν_{max} (neat)/cm^{-1}:** 3065, 2954, 2865, 1568, 1475, 1428, 1361, 1255, 1115, 1072, 1043, 867, 780, 689.

2-(4-Bromophenyl)-4-fluoropyrans *syn*-5g and *anti*-5g.

Products ***syn*-5g** and ***anti*-5g** were prepared according to the general procedure outlined for the oxa-Prins reaction from 4-bromobenzaldehyde (92 mg, 0.5 mmol), but-3-en-1-ol **3** (45 µL, 0.5 mmol) and boron trifluoride (65 µL, 0.5 mmol).

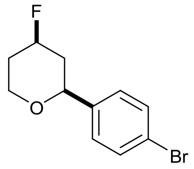

***syn*-5g**

Product ***syn*-5g** was isolated after purification on silica gel (hexane/diethyl ether, 8/2) as a white solid (92 mg, 71%, microwave: 71 mg, 55%).

NMR: [1]H, 300 MHz (δ in ppm): 7.53-7.45 (2H, m, ar), 7.26-7.20 (2H, m, ar), 4.81 (1H, dtt, *J*= 48.9 Hz, 11.0 Hz, 5.1 Hz, C*H*F), 4.29 (1H, dt, *J*= 11.6 Hz, 1.9 Hz, OC*H*CH_2), 4.21 (1H, dtd, *J*= 12.0 Hz, 5.8 Hz, 1.8 Hz, OC*H*HCH_2), 3.56 (1H, tt, *J*= 12.3 Hz, 1.9 Hz, OC*H*HCH_2), 2.32 (1H, dtt, *J*= 12.3 Hz, 4.8 Hz, 2.1 Hz, OCHC*H*H), 2.18-2.08 (1H, m, OCH_2C*H*H), 1.85 (1H, tddd, *J*= 12.5 Hz, 11.0 Hz, 9.9 Hz, 5.1 Hz, OCH_2C*H*H), 1.79-1.62 (1H, m, OCHC*H*H); **[13]C, 75 MHz (δ in ppm):** 140.3 (Cq, ar), 131.5 (Cq, ar), 127.5 (CH, ar), 121.6 (CH, ar), 89.1 (CH, d, *J*= 177.6 Hz, *C*HF), 77.0 (CH, d, *J*= 11.5 Hz, O*C*HCH_2), 65.4 (CH_2, d, *J*= 12.0 Hz, O*C*H_2CH_2), 40.4 (CH_2, d, *J*= 17.0 Hz, OCH*C*H_2), 32.8 (CH_2, d, *J*= 18.3 Hz, OCH_2*C*H_2); **[19]F, 282 MHz (δ in ppm):** -170.1 to -170.7 (m); **HRMS m/z:** $[MH, -HF]^+$: 239.0072, 241.0057, calculated 239.0072, 241.0051; **Mp:** 36-38 °C; **IR: ν_{max} (neat)/cm^{-1}:** 3082, 2959, 2856, 1588, 1489, 1453, 1362, 1247, 1158, 1089, 978, 822, 589.

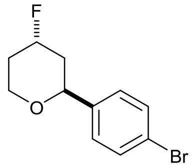

***anti*-5g**

Product ***anti*-5g** was isolated after purification on silica gel (hexane/diethyl ether, 8/2) as a colorless oil (18 mg, 14%, microwave: 26 mg, 20%).

NMR: [1]H, 300 MHz (δ in ppm): 7.51-7.48 (2H, m, ar), 7.26-7.21 (2H, m, ar), 5.08 (1H, dquint, *J*= 48.1 Hz, 2.8 Hz, C*H*F), 4.72 (1H, dd, *J*= 11.8 Hz, 2.3 Hz, OC*H*CH_2), 4.04-3.98 (2H, m, OC*H*$_2CH_2$), 2.15 (1H, dddt, *J*= 14.6 Hz, 11.1 Hz, 3.3 Hz, 2.2 Hz,m OCHC*H*H), 2.06-1.63 (3H, m, OCH_2C*H*$_2$, OCHC*H*H); **[13]C, 75 MHz (δ in ppm):** 145.9 (Cq, ar), 136.0 (Cq, ar),131.5 (CH, ar), 127.4 (CH, ar), 86.5 (CH, d, *J*= 169.1 Hz, *C*HF), 73.4 (CH, O*C*HCH_2), 63.0 (CH_2, O*C*H_2CH_2), 38.7 (CH_2, d, *J*= 20.8 Hz,

OCHC*H*$_2$), 30.5 (CH_2, d, *J*= 21.5 Hz, OCH_2C*H*$_2$); **^{19}F, 282 MHz (δ in ppm):** -185.8 to -186.6 (m); **HRMS m/z:** $[MH, -HF]^+$: 239.0077; 241.0052, calculated 239.0072, 241.0051; **Mp:** 39-41 °C; **IR: v_{max} (neat)/cm^{-1}:** 3079, 2918, 2850, 1580, 1475, 1360, 1255, 1202, 1146, 1069, 1007, 814.

2-(4-Methoxyphenyl)-4-fluoropyrans *syn*-5i and *anti*-5i.

Products ***syn*-5i** and ***anti*-5i** were prepared according to the general procedure outlined for the oxa-Prins reaction from 4-methoxybenzaldehyde (60 µL, 0.5 mmol), but-3-en-1-ol **3** (45 µL, 0.5 mmol) and boron trifluoride (65 µL, 0.5 mmol). The product was isolated as a white solid as a mixture of diastereoisomers after purification on silica gel (hexane/diethyl ether, 8/2) (17 mg, 16%, mixture ratio *syn*/*anti* 2.5/1, microwave: 39 mg, 37%, mixture ratio *syn*/*anti* 1/1.9).

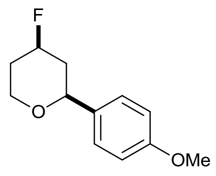

***syn*-5i**

Product ***syn*-5i**, identified from the mixture of diastereoisomers:

NMR: ^{1}H, 300 MHz (δ in ppm): 7.33-7.25 (2H, m, ar), 6.93-6.85 (2H, m, ar), 4.81 (1H, dtt, *J*= 49.2 Hz, 11.0 Hz, 5.0 Hz, C*H*F), 4.28 (1H, dt, *J*= 11.5 Hz, 2.0 Hz, OC*H*CH$_2$), 4.20 (1H, dtd, J= 12.0 Hz, 5.4 Hz, 1.7 Hz, OC*H*HCH$_2$), 3.81 (3H, s, OCH_3), 3.57 (1H, tt, *J*= 12.2 Hz, 2.0 Hz, OC*H*HCH$_2$), 2.31 (1H, dtt, *J*= 12.4 Hz, 5.0 Hz, 2.1 Hz, OCHC*H*H), 2.21-2.07 (1H, m, OCH_2C*H*H), 2.07-1.71 (2H, m, OCH_2C*H*H, OCHC*H*H); **^{13}C, 75 MHz (δ in ppm):** 158.0 (Cq, ar), 134.3 (Cq, ar), 127.2 (CH, ar), 113.8 (CH, ar), 89.4 (CH, d, *J*= 176.6 Hz, *C*HF), 77.6 (CH, O*C*HCH$_2$), 65.4 (CH_2, d, *J*= 12.4 Hz, O*C*H$_2$CH$_2$), 55.2 (CH_3, OCH3), 40.3 (CH_2, d, *J*= 17.1 Hz, OCH*C*H$_2$), 32.9 (CH_2, d, *J*= 16.2 Hz, OCH_2*C*H$_2$); **^{19}F, 282 MHz (δ in ppm):** -169.9 to -170.3 (m); **HRMS m/z:** $[MH]^+$: 211.1133, calculated 211.1134; **IR of the mixture: v_{max} (neat)/cm^{-1}:** 3079, 2932, 2858, 1518, 1450, 1347, 1295, 1169, 1081, 951, 854, 586.

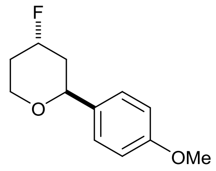

***anti*-5i**

Product ***anti*-5i**, identified from the mixture of diastereoisomers:

NMR: ^{1}H, 300 MHz (δ in ppm): 7.33-7.25 (2H, m, ar), 6.93-6.85 (2H, m, ar), 5.92 (1H, dquint, *J*= 48.0 Hz, 2.8 Hz, C*H*F), 4.71 (1H, dd, *J*= 11.6 Hz, 2.2 Hz, OC*H*CH_2), 4.04-3.97 (2H, m, O$CH_2$$CH_2$), 3.81 (3H, s, OCH_3), 2.21-2.07 (1H, m, OCHC*H*H), 2.07-1.71 (3H, m, OCH_2CH_2, OCHC*H*H); **^{13}C, 75 MHz (δ in ppm):** 159.0 (Cq, ar), 134.3 (Cq, ar), 127.1 (CH, ar), 113.8 (CH, ar), 86.9 (CH, d, *J*= 168.6 Hz, *C*HF), 73.7 (CH, O*C*HCH_2), 63.1 (CH_2, O*C*H_2CH_2), 55.2 (CH_3, OCH_3), 38.4 (CH_2, d, *J*= 20.7 Hz, OCH*C*H_2), 30.6 (CH2, d, *J*= 20.7 Hz, OCH_2*C*H_2); **^{19}F, 282 MHz (δ in ppm):** -185.8 to -186.4 (m); **HRMS m/z:** $[MH]^+$: 211.1133, calculated 211.1134; **IR of the mixture: ν_{max} (neat)/cm^{-1}:** 3079, 2932, 2858, 1518, 1450, 1347, 1295, 1169, 1081, 951, 854, 586.

2-Pentyl-4-fluoropyrans *syn*-5k and *anti*-5k.

Products ***syn*-5k** and ***anti*-5k** were prepared according to the general procedure outlined for the oxa-Prins reaction from hexanal (61 µL, 0.5 mmol), but-3-en-1-ol (45 µL, 0.5 mmol) and boron trifluoride (65 µL, 0.5 mmol).

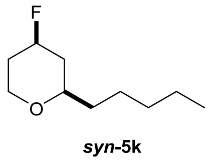

***syn*-5k**

Product ***syn*-5k** was isolated after purification on silica gel (hexane/diethyl ether, 8/2) as a pale yellow oil (42 mg, 48%, from microwave: 51 mg, 58%, at -20 °C: 57 mg, 66%).

NMR: ^{1}H, 400 MHz (δ in ppm): 4.63 (1H, dtt, *J*= 49.2 Hz, 11.0 Hz, 5.1 Hz, C*H*F), 4.02 (1H, dtd, *J*= 11.7 Hz, 5.7 Hz, 1.8 Hz, OC*H*HCH$_2$), 3.35 (1H, tt, *J*= 12.3 Hz, 1.6 Hz, OC*H*HCH$_2$), 3.23 (1H, dddt, *J*= 11.3 Hz, 7.1 Hz, 4.7 Hz, 1.8 Hz, OC*H*CH$_2$), 2.07 (1H, dtt, *J*= 12.1 Hz, 5.1 Hz, 1.8 Hz, OCHC*H*H), 2.03-1.96 (1H, m, OCH$_2$C*H*H), 1.76-1.62 (1H, tddd, *J*= 12.3 Hz, 11.0 Hz, 9.9 Hz, 5.1 Hz, OCH$_2$C*H*H), 1.61-1.20 (9H, m, OCHC*H*H, 4xCH$_2$ pentyl), 0.88 (3H, t, *J*= 6.7 Hz, CH$_3$ pentyl); **^{13}C, 100 MHz (δ in ppm):** 89.3 (CH, d, *J*= 176.2 Hz, *C*HF), 75.7 (CH, d, *J*= 10.9 Hz, O*C*HCH$_2$), 64.9 (CH$_2$, d, *J*= 11.4 Hz, O*C*H$_2$CH$_2$), 38.7 (CH$_2$, d, *J*= 17.1 Hz, OCH*C*H$_2$), 36.0 (CH$_2$, CH$_2$ pentyl), 33.1 (CH$_2$, d, *J*= 17.4 Hz, OCH$_2$*C*H$_2$), 31.7 (CH$_2$, CH$_2$ pentyl), 25.1 (CH$_2$, CH$_2$ pentyl), 22.5 (CH$_2$, CH$_2$ pentyl), 14 (CH$_3$, CH$_3$ pentyl); **^{19}F, 376 MHz (δ in ppm):** -169.7 to -170.0 (m); **HRMS m/z:** [MH, -HF]$^{+}$**:** 155.1435, calculated 155.1436; **IR: ν_{max} (neat)/cm^{-1}:** 2955, 2931, 2858, 1456, 1367, 1163, 1085, 1005.

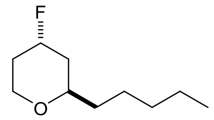

***anti*-5k**

Product ***anti*-5k** was isolated after purification on silica gel (hexane/diethyl ether, 8/2) as a colorless oil (20 mg, 23%, microwave: 26 mg, 30%).

NMR: ^{1}H, 400 MHz (δ in ppm): 4.99 (1H, dquint, *J*= 48.1 Hz, 2.7 Hz, C*H*F), 3.88-3.76 (2H, m, OC*H*$_2$CH$_2$), 3.65 (1H, dddd, *J*= 11.6 Hz, 7.0 Hz, 4.4 Hz, 2.1 Hz, OC*H*CH$_2$), 1.90-1.70 (2H, m, OCHC*H*H, OCH$_2$C*H*H), 1.59-1.21 (10H, m, OCH$_2$C*H*H, OCHC*H*H, 4xCH$_2$ pentyl) 0.89 (3H, t, *J* = 7.13 Hz, CH$_3$ pentyl); **^{13}C, 100 MHz (δ in ppm):** 86.9 (CH, d, *J*= 169.3 Hz, *C*HF), 71.9 (CH, O*C*HCH$_2$), 62.5 (CH$_2$, O*C*H$_2$CH$_2$), 36.8 (CH$_2$, d, *J*= 19.6 Hz, OCH*C*H$_2$), 36.1 (CH$_2$, CH$_2$ pentyl), 31.8 (CH$_2$, CH$_2$ pentyl), 31.0 (CH$_2$, d, *J*= 19.6 Hz, OCH$_2$*C*H$_2$), 25.0 (CH$_2$, CH$_2$ pentyl), 22.6 (CH$_2$, CH$_2$ pentyl), 14.1 (CH$_3$, CH$_3$ pentyl); **^{19}F, 376 MHz (δ in ppm):** -185.2 to -185.8 (m); **HRMS m/z:** [MH, -HF]$^{+}$: 155.1435, calculated 155.1436; **IR: ν_{max} (neat)/cm^{-1}:** 2934, 2927, 2858, 1462, 1365, 1200, 1146, 1073, 1005.

2-Phenyl-4-fluoropyrans *syn*-5m and *anti*-5m.

Products ***syn*-5m** and ***anti*-5m** were prepared according to the general procedure outlined for the oxa-Prins reaction from freshly distilled benzaldehyde (50 µL, 0.5 mmol), but-3-en-1-ol **99** (45 µL, 0.5 mmol) and boron trifluoride (65 µL, 0.5 mmol).

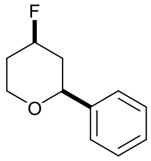

***syn*-5m**

Product ***syn*-5m** was isolated after purification on silica gel as a single diastereoisomer (hexane/diethyl ether, 8/2) as a colorless oil (19 mg, 21%, microwave: 8 mg, 9%, at -20 °C: 54 mg, 59%).

NMR: ^{1}H, 400 MHz (δ in ppm): 7.30-7.16 (5H, m, ar), 4.74 (1H, dtt, *J*= 49.1 Hz, 10.9 Hz, 5.0 Hz, C*H*F), 4.23 (1H, dt, *J*= 11.6 Hz, 1.8 Hz, OC*H*CH_2), 4.13 (1H, dtd, *J*= 11.9 Hz, 5.7 Hz, 1.8 Hz, OC*H*HCH_2), 3.48 (1H, tt, *J*= 12.2 Hz, 1.8 Hz, OC*H*HCH_2), 2.26 (1H, dtt, *J*= 12.3 Hz, 4.9 Hz, 2.1 Hz, OCHC*H*H), 2.04 (1H, dddt, *J*= 12.3 Hz, 6.9 Hz, 4.3 Hz, 2.0 Hz, OCH_2C*H*H), 1.86-1.60 (2H, m, OCHC*H*H, OCH_2C*H*H); **^{13}C, 100 MHz (δ in ppm):** 141.2 (Cq, ar), 128.4 (CH, ar), 127.8 (CH, ar), 125.8 (CH, ar), 89.3 (CH, d, *J*= 176.9 Hz, *C*HF), 77.8 (CH, d, *J*= 11.3 Hz, O*C*HCH_2), 65.4 (CH_2, d, *J*= 11.9 Hz, O*C*$H_2$$CH_2$), 40.5 ($CH_2$, d, *J*= 17.0 Hz, O$CH_2$*C*$H_2$), 32.9 ($CH_2$, d, *J*= 17.6 Hz, OCH*C*H_2); **^{19}F, 376 MHz (δ in ppm):** -169.9 to -170.4 (m); **HRMS m/z:** $[MNa]^+$: 203.0843, calculated 203.0848; **IR: v_{max} (neat)/cm^{-1}:** 3064, 3032, 2960, 2853, 1494, 1453, 1374, 1249, 1158, 1081, 980, 757, 699.

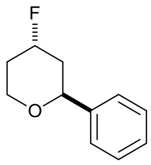

***anti*-5m**

Product ***anti*-5m** was isolated as a mixture of diastereoisomers with **101r** after purification on silica gel (hexane/diethyl ether, 8/2) as a colorless oil (32 mg, 35%, mixture ratio *syn*/*anti* 2.4/1, microwave: 47 mg, 52%, mixture ratio *syn*/*anti* 2.7/1).

From the mixture of diastereoisomers :

NMR: ^{1}H, 300 MHz (δ in ppm): 7.29-7.17 (5H, m, ar), 5.01 (1H, dquint, *J*= 48.1 Hz, 3.2 Hz, C*H*F), 4.67 (1H, dd, *J*= 11.9 Hz, 2.6 Hz, OC*H*CH_2), 3.95-3.91 (2H, m, OC$H_2$$CH_2$), 2.14-2.00 (1H, m, OCHC*H*H), 1.98-1.62 (3H, m, OCH_2CH_2, OCHC*H*H); **^{13}C, 75 MHz (δ in ppm):** 142.2 (Cq, ar), 128.5 (CH, ar), 127.6 (CH, ar), 125.8 (CH, ar), 86.9 (CH, d, *J*= 168.7 Hz, *C*HF), 74.1 (CH, O*C*HCH_2), 63.1 (CH_2, O$CH_2$$CH_2$), 38.6 ($CH_2$, d, *J*= 21.0 Hz, OCH$CH_2$), 30.6 ($CH_2$, d, *J*= 22.4 Hz, O$CH_2$$CH_2$); **^{19}F, 282 MHz (δ in ppm):** -185.9 to -186.4 (m); **HRMS m/z:** $[MNa]^+$: 203.0846, calculated 203.0848; **IR of the mixture of diastereoisomers: ν_{max} (neat)/cm^{-1}:** 3064, 3032, 2960, 2853, 1494, 1453, 1374, 1249, 1158, 1081, 980, 757, 699.

3-Fluoro-5-phenylpentan-1-ol 7.

Pd on charcoal (4 mg) was added to a solution of 2-phenyl-4-fluoropyran **5m** (47 mg, 0.26 mmol) in acetic acid (4 mL) and perchloric acid (40 µL, 60% in water). The mixture was stirred under a hydrogen atmosphere for 16 h. The mixture was then filtered through celite and the product was extracted with ethyl acetate. The organic layer was dried, filtered and concentrated. The title compound was obtained after purification on silica (hexane/diethyl ether 7/3) as a colorless oil (41 mg, 70%).

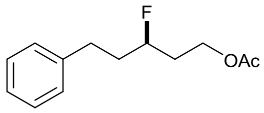

7

NMR: ^{1}H, 400 MHz (δ in ppm): 7.37-7.28 (2H, m, ar), 7.26-7.17 (3H, m, ar), 4.63 (1H, dtt, *J*= 49.5 Hz, 8.6 Hz, 3.6 Hz, C*H*F), 4.30-4.13 (2H, m, CH_2CH_2OH), 2.84 (1H, ddd, *J*= 13.8 Hz, 9.9 Hz, 5.5 Hz, PhC*H*HCH_2), 2.72 (1H, ddd, *J*= 13.8 Hz, 9.4 Hz, 7.1 Hz, PhC*H*HCH_2), 2.12-1.75 (7H, m, PhCH_2CH_2, HOCH_2CH_2, C=OCH_3); **^{13}C, 100 MHz (δ in ppm):** 171.0 (Cq, *C*=OCH_3), 141.2 (Cq, ar), 128.5 (CH, ar), 128.4 (CH, ar), 126.0 (CH, ar), 90.2 (CH, d, *J*= 168.6 Hz, *C*HF), 60.6 (CH, d, *J*= 4.7 Hz, CH_2*C*H_2OH), 36.9 (CH_2, d, *J*= 21.0 Hz, HOCH_2*C*H_2), 34.2 (CH_2, d, *J*= 21.0 Hz, PhCH_2*C*H_2), 31.2 (CH_2, d, *J*= 4.7 Hz, Ph*C*$H_2$$CH_2$), 20,9 ($CH_3$, C=O*C*$H_3$); **^{19}F, 376**

MHz (δ in ppm): -185.1 to -185.7 (m); **HRMS m/z:** $[MNa]^+$: 247.1109, calculated 247.1110; **IR: v_{max} (neat)/cm^{-1}:** 3027, 2932, 1739, 1385, 1365, 1248, 1046, 746, 700.0.

***trans*-2-Vinylcyclohexanol 8**.

To a solution of cyclohexene oxide **10** (500 µL, 5 mmol) in diethyl ether (2 mL) at -20 °C, was added $CuBr.Me_2S$ (102 mg, 0.5 mmol) and vinylmagnesium bromide (6 mL, 6 mmol). After 10h, the mixture was hydrolysed with a solution of saturated ammonium chloride and the layers separated. The aqueous layer was extracted with diethyl ether. The organic layers were dried, concentrated and purified on silica gel. Alcohol **8** was obtained as a colorless oil (447 mg, 71%)

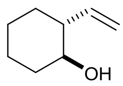

8

NMR: ^{1}H, 400 MHz (δ in ppm): 5.68 (1H, ddd, *J*= 17.2 Hz, 10.2 Hz, 8.7 Hz, C*H*=CH_2), 5.16 (1H, ddd, *J*= 17.2 Hz, 1.9 Hz, 0.7 Hz, CH=CH_2), 5.12 (1H, dd, *J*= 10.2 Hz, 1.9 Hz, CH=CH_2), 3.24 (1H, td, *J*= 10.0 Hz, 4.4 Hz, C*H*OH), 2.06-1.98 (1H, m, CH*H*CHOH), 1.96-1.84 (1H, m, C*H*CH=CH_2), 1.79-1.70 (2H, m, CH*H*CH_2CHOH or CH*H*CH_2CH_2CHOH, CH*H*CHCH=CH_2), 1.69-1.64 (1H, m, CH*H*CH_2CHOH or CH*H*CH_2CH_2CHOH), 1.31-1.15 (4H, CH*H*CHCH=CH_2, CH*H*CH_2CHOH, CH*H*CH_2CH_2CHOH, CH*H*CHOH); **^{13}C, 100 MHz (δ in ppm):** 140.8 (CH, *C*H=CH_2), 116.7 (CH_2, CH=*C*H_2), 72.7 (CH, *C*HOH), 51.2 (CH, *C*HCH=CH_2), 33.8 (CH_2, *C*H_2CHOH), 31.1 (CH_2, *C*H_2CHCH=CH_2), 25.1, 24.7 (CH_2, *C*H_2CH_2CHOH, *C*$H_2CH_2CH_2$CHOH); **LRMS m/z:** $[MNa]^+$: 149.1, calculated 149.1; **IR: v_{max} (neat)/cm^{-1}:** 3384, 2918, 2851, 1697, 1446, 1303, 1200, 1057.

4-Fluoro-2-phenyloctahydrochromene 9a.

Product **9a** was prepared according to the general procedure outlined for the oxa-Prins reaction from benzaldehyde (102 µL, 1.0 mmol), 2-vinylcyclohexanol **8** (126 mg, 1.0 mmol) and boron trifluoride (126 µL, 0.1 mmol). The product was isolated after purification on silica gel (hexane/diethyl ether, 8/2) as a pale yellow oil (69 mg, 59%).

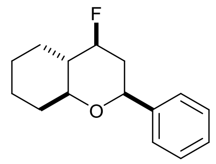

9a

NMR: ^{1}H, 300 MHz (δ in ppm): 7.39-7.33 (4H, m, ar), 7.31-7.27 (1H, m, ar), 4.46 (1H, dtd, *J*= 49.9 Hz, 10.4 Hz, 5.0 Hz, C*H*F), 4.44 (1H, dt, *J*= 11.82 Hz, 2.0 Hz, OC*H*ArCH$_2$), 3.16 (1H, tdd, *J*= 10.4 Hz, 4.2 Hz, 1.5 Hz, OC*H*CH$_2$), 2.35 (1H, dtd, *J*= 12.4 Hz, 4.7 Hz, 2.0 Hz, OCHArC*H*HCHF), 2.21 (1H, ddt, *J*= 13.3 Hz, 5.6 Hz, 3.0 Hz, CHFCHC*H*HCH$_2$), 2.05 (1H, m, OCHC*H*HCH$_2$), 1.91-1.81 (2H, m, CHFC*H*$_2$CHOAr and CHFCHCH$_2$C*H*$_2$), 1.80-1.72 (2H, m, CHFCHCH$_2$C*H*$_2$), 1.60-1.42 (2H, m, OCHC*H*$_2$CH$_2$, CHFC*H*CH$_2$), 1.40-1.24 (2H, m, OCHCH$_2$C*H*$_2$), 1.13-0.96 (1H, m, CHFCHC*H*HCH$_2$), 1.10-1.00 (1H, m, CHFCHC*H*$_2$CH$_2$); **^{13}C, 75 MHz (δ in ppm):** 136.3 (Cq, ar), 128.5 (CH, ar), 127.7 (CH, ar), 126.0 (CH, ar), 93.6 (CH, d, *J*= 177.7 Hz, *C*HF), 78.7 (CH, d, *J*= 8.8 Hz, O*C*HCH$_2$), 77.1 (CH, d, *J*= 12.5 Hz, O*C*HAr), 47.9 (CH, d, *J*= 16.9 Hz, CHF*C*HCH$_2$), 40.4 (CH$_2$, d, *J*= 17.4 Hz, CHF*C*H$_2$CHAr), 32.0 (CH$_2$, OCH*C*H$_2$CH$_2$), 26.9 (CH$_2$, CHFCH*C*H$_2$), 24.9 (CH$_2$, CHFCHCH$_2$*C*H$_2$CH$_2$ or CHFCHCH$_2$CH$_2$*C*H$_2$), 24.7 (CH$_2$, CHFCHCH$_2$*C*H$_2$CH$_2$ or CHFCHCH$_2$CH$_2$*C*H$_2$); **^{19}F, 282 MHz (δ in ppm):** -179.8 to -180.1 (m); **HRMS m/z:** [MNa]$^+$: 257.1318, calculated 257.1319; **IR: ν_{max} (neat)/cm^{-1}:** 3027, 2920, 2851, 1449, 1372, 1278, 1183, 1123, 1072, 1034, 997, 756, 698.

4-Fluoro-2-(4-nitrophenyl)octahydrochromene 9b.

Product **9b** was prepared according to the general procedure outlined for the oxa-Prins reaction from 4-nitrobenzaldehyde (121 mg, 0.87 mmol), 2-vinyl-cyclohexanol **8** (110 mg, 0.87 mmol) and boron trifluoride (110 µL, 0.87 mmol). The title compound was isolated after purification on silica gel (hexane/diethyl ether, 8/2) as a white solid (80 mg, 57%).

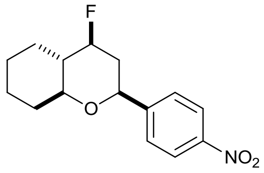

9b

NMR: ^{1}H, 300 MHz (δ in ppm): 8.20 (2H, m, ar), 7.54 (2H, m, ar), 4.55 (1H, m, OC*H*ArCH$_2$), 4.47 (1H, dtd, *J*= 49.6 Hz, 10.4 Hz, 4.9 Hz, C*H*F), 3.18 (1H, tdd, *J*= 10.3 Hz, 4.1 Hz, 1.4 Hz, OC*H*CH$_2$), 2.38 (1H, dtd, *J*= 12.3 Hz, 4.5 Hz, 2.3 Hz, OCHArC*H*HCHF), 2.21 (1H, ddt, *J*= 13.2 Hz, 5.3 Hz, 3.0 Hz, CHFCHC*H*HCH$_2$), 2.10-2.00 (1H, m, OCHC*H*HCH$_2$), 1.91-1.81 (1H, m, OCHCH$_2$C*H*HCH$_2$ or OCHCH$_2$CH$_2$C*H*H), 1.81-1.68 (2H, m, OCHArC*H*HCHF, OCHCH$_2$C*H*HCH$_2$ or OCHCH$_2$CH$_2$C*H*H), 1.60-1.39 (1H, m, OCHC*H*, OCHC*H*HCH$_2$), 1.39-1.18 (2H, m, OCHCH$_2$C*H*HCH$_2$, OCHCH$_2$CH$_2$C*H*H), 1.13-0.96 (1H, m, CHFCHC*H*HCH$_2$); **^{13}C, 75 MHz (δ in ppm):** 148.7 (Cq, ar), 147.3 (Cq, ar), 126.5 (CH, ar), 123.6 (CH, ar), 92.8 (CH, d, *J*= 178.8 Hz, *C*HF), 78.7 (CH, d, *J*= 9.3 Hz, O*C*HCH), 75.7 (CH, d, *J*= 12.0 Hz, O*C*HArCH$_2$), 47.7 (CH, d, *J*= 17.4 Hz, OCH*C*H), 40.4 (CH$_2$, d, *J*= 18.5 Hz, OCHAr*C*H$_2$CHF), 31.9 (CH$_2$, CHFCH*C*H$_2$), 26.7 (CH$_2$, OCH*C*H$_2$CH$_2$), 24.8 (CH$_2$, OCHCH$_2$*C*H$_2$CH$_2$ or OCHCH$_2$CH$_2$*C*H$_2$), 24.5 (CH$_2$, OCHCH$_2$*C*H$_2$CH$_2$ or OCHCH$_2$CH$_2$*C*H$_2$); **^{19}F, 282 MHz (δ in ppm):** -181.6 to -182.0 (m); **HRMS m/z:** [MNa]$^{+}$: 302.1170, calculated 302.1168; **Mp:** 77-79 °C; **IR: ν_{max} (neat)/cm^{-1}:** 3080, 2932, 2858, 1604, 1519, 1348, 1169, 1081, 1049, 854.

3-Ethyl-4-fluoro-2-(4-nitrophenyl)tetrahydropyran 12a.

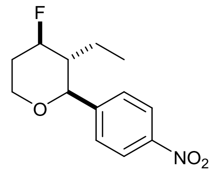

12a

Pyran **12a** was prepared according to the general procedure outlined for the oxa-Prins reaction from 4-nitrobenzaldehyde (151 mg, 1 mmol), *(E)*-hex-3-en-1-ol **11a** (118 µL, 1 mmol) and boron trifluoride (127 µL, 1 mmol). The title compound was obtained after purification on silica gel (hexane/diethyl ether, 8/2) as a white solid (67 mg, 53%).

NMR: ^{1}H, 300 MHz (δ in ppm): 8.25-8.17 (2H, m, ar), 7.56-7.49 (2H, m, ar), 4.64 (1H, dtd, *J*= 49.2 Hz, 10.6 Hz, 4.9 Hz, C*H*F), 4.19-4.08 (2H, m , OC*H*CH, OC*H*HCH$_2$), 3.57 (1H, tdd, *J*= 12.5 Hz, 2.1 Hz, 1.5 Hz, OC*H*HCH$_2$), 2.19 (1H, dddd, *J*= 12.2 Hz, 6.8 Hz, 4.9 Hz, 2.0 Hz, OCH$_2$C*H*H), 2.02 (1H, m, OCH$_2$C*H*H), 1.79 (1H, qt, *J*= 9.7 Hz, 4.9 Hz, OCHC*H*), 1.46-1.31 (1H, m, CHCH*H*CH$_3$), 1.28-1.17 (1H, m, CHCH*H*CH$_3$), 0.74 (3H, td, *J*= 7.6 Hz, 0.9 Hz, CH$_2$C*H$_3$*); **^{13}C, 75 MHz (δ in ppm):** 147.7 (Cq, ar), 146.9 (Cq, ar), 128.3 (CH, ar), 123.6 (CH, ar), 91.8 (CH, d, *J*= 178.8 Hz, *C*HF), 81.5 (CH, d, *J*= 9.9 Hz, O*C*HCH), 65.4 (CH$_2$, d, *J*= 12.9 Hz, O*C*H$_2$CH$_2$), 49.0 (CH, d, *J*= 17.4 Hz, OCH*C*H), 32.9 (CH$_2$, d, *J*= 18.5 Hz, OCH$_2$*C*H$_2$), 20.0 (CH$_2$, *C*H$_2$CH$_3$), 10.5 (CH$_3$, CH$_2$*C*H$_3$); **^{19}F, 282 MHz (δ in ppm):** -176.0 to -176.5 (m); **HRMS m/z:** [MNa]$^+$: 276.1015, calculated 276.1012; **Mp:** 81-83 °C; **IR: ν_{max} (neat)/cm^{-1}:** 3110, 3080, 2966, 2858, 1605, 1522, 1347, 1198, 1155, 1088, 1025, 852, 814.

3-Ethyl-4-fluoro-2-(4-nitrophenyl)tetrahydropyran 12b.

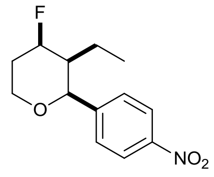

12b

Pyran **12b** was prepared according to the general procedure outlined for the oxa-Prins reaction from 4-nitrobenzaldehyde (151 mg, 1 mmol), *(Z)*-hex-3-en-1-ol **11b** (118 µL, 1 mmol) and boron trifluoride (127 µL, 1 mmol). The title compound was obtained after purification on silica gel (hexane/diethyl ether, 8/2) as a white solid (68 mg, 54%).

NMR: ^{1}H, 300 MHz (δ in ppm): 8.25-8.17 (2H, m, ar), 7.55-7.46 (2H, m, ar), 4.96 (1H, ddt, *J*= 48.6 Hz, 11.6 Hz, 5.1 Hz, C*H*F), 4.51 (1H, t, *J*= 2.3 Hz, OC*H*CH), 4.21 (1H, dtd, *J*= 11.6 Hz, 6.1 Hz, 1.5 Hz, OC*H*HCH_2), 3.56 (1H, dddd, *J*= 12.3 Hz, 12.0 Hz, 2.9 Hz, 1.5 Hz, OC*H*HCH_2), 2.23 (1H, m, OCHC*H*), 2.02 (1H, dddd, *J*= 24.5 Hz, 12.6 Hz, 8.2 Hz, 5.5 Hz, OCH_2C*H*H), 1.95-1.85 (1H, m, OCH_2C*H*H), 1.50 (1H, dqd, *J*= 14.6 Hz, 7.6 Hz, 4.9 Hz, OCHCHCH*H*CH_3), 1.21 (1H, dqd, *J*= 14.6 Hz, 7.6 Hz, 5.8 Hz, OCHCHCH*H*CH_3), 0.47 (3H, dt, *J*= 7.6 Hz, 0.8 Hz, OCHCHCH_2CH_3); **^{13}C, 75 MHz (δ in ppm):** 147.9 (Cq, ar), 146.0 (Cq, ar), 126.3 (CH, ar), 123.4 (CH, ar), 92.4 (CH, d, *J*= 183.0 Hz, *C*HF), 79.0 (CH, d, *J*= 9.4 Hz, O*C*HCH), 65.2 (CH_2, d, *J*= 11.9 Hz, OCH_2CH_2), 47.0 (CH, d, *J*= 16.2 Hz, OCH*C*H), 27.2 (CH_2, d, *J*= 18.7 Hz, OCH_2CH_2), 14.7 (CH_2, d, *J*= 1.7 Hz, *C*H_2CH_3), 14.4 (CH_3, d, *J*= 1.8 Hz, CH_2CH_3); **^{19}F, 282 MHz (δ in ppm):** -176.9 to -177.3 (m); **HRMS m/z:** $[MNa]^+$: 276.1019, calculated 276.1012; **Mp:** 81-83 °C; **IR: ν_{max} (neat)/cm^{-1}:** 3112, 3080, 2966, 2876, 1601, 1519, 1346, 1171, 1104, 1072, 1026, 856.

***N*-Tosyl-3-butenylamine 13.**

1-Bromobut-3-ene (1.01 ml, 10 mmol) was added to a solution of tosylamine (1.71g, 10 mmol), and potassium carbonate (1.65 g, 12 mmol) in acetone (100 mL). After 4h at reflux, the mixture was concentrated under vacuum, and water and diethyl ether were added. The layers were separated and the aqueous layer extracted with diethyl ether. The organic layers were dried, filtered and concentrated. The title compound was obtained after purification on silica (hexane/diethyl ether 6/4) as a pale yellow oil (922 mg, 41%).

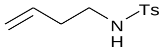

13

NMR: ^{1}H, 300 MHz (δ in ppm): 7.80-7.70 (2H, m, ar), 7.35-7.28 (2H, m, ar), 5.62 (1H, ddt, *J*= 16.9 Hz, 10.3 Hz, 6.8 Hz, CH_2=C*H*), 5.08-4.97 (2H, m, CH_2=CH), 4.76 (1H, t broad, *J*= 5.8 Hz, N*H*), 3.00 (2H, td, *J*= 6.8 Hz, 5.8 Hz, CH_2CH_2NH), 2.42 (3H, m, ArCH_3), 2.19 (2H, qt, *J*= 6.8 Hz, 1.4 Hz, CHC$H_2$$CH_2$NH); **^{13}C, 100 MHz (δ in ppm):** 143.2 (Cq, ar), 138.1 (Cq, ar), 130.0 (CH, ar), 127.1 (CH, ar), 136.2 (CH, CH_2=*C*H), 116.4 (CH_2, *C*H_2=CH), 48.1 (CH_2, *C*H_2NHTs), 34.3 (CH_2, CH_2=CH*C*H_2), 21.5 (CH_3, arCH_3); **LRMS m/z:** $[MH]^+$: 225.1, calculated 225.1; **IR: v_{max} (neat)/cm^{-1}:** 3283, 3075, 2974, 2924, 2868, 1641, 1597, 1493, 1323, 1158, 1090, 1085, 990, 920, 813.

***N*-(Tosyl)-2-(4-nitrophenyl)-4-fluoropiperidines *syn*-14a and *anti*-14a.**

Piperidines ***syn*-14a** and ***anti*-14a** were prepared according to the general procedure outlined for the aza-Prins reaction from 4-nitrobenzaldehyde (76 mg, 0.5 mmol), *N*-tosyl-3-butenylamine **13** (112mg, 0.5 mmol) and boron trifluoride (65 µL, 0.5 mmol).

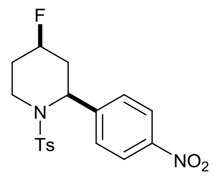

***syn*-14a**

Product ***syn*-14a** was isolated after purification on silica gel (hexane/diethyl ether, 7/3) as a colorless oil (59 mg, 31%, microwave: 62 mg, 33%).

NMR: ^{1}H, 400 MHz (δ in ppm): 8.28-8.18 (2H, m, ar), 7.79-7.73 (2H, m, ar), 7.58-7.52 (2H, m, ar), 7.40-7.34 (2H, m, ar), 5.42 (1H, s broad, NC*H*CH_2), 4.54 (1H, dtt, *J*= 48.3 Hz, 10.4 Hz, 4.5 Hz, C*H*F), 4.03-3.94 (1H, m, NC*H*HCH_2), 3.10-2.99 (1H, m, NC*H*HCH_2), 2.68-2.58 (1H, m, NCHC*H*H), 2.47 (3H, s, arCH_3), 1.98-1.86 (1H, m, NCH_2C*H*H), 1.79 (1H, dddd, *J*= 13.8 Hz, 10.8 Hz, 8.6 Hz, 5.7 Hz, NCHC*H*H), 1.49 (1H, ttd, J= 12.5 Hz, 10.2 Hz, 4.8 Hz, NCH_2C*H*H); **^{13}C, 100 MHz (δ in ppm):** 147.3 (Cq, ar), 145.8 (Cq, ar), 144.1 (Cq, ar), 137.2 (Cq, ar), 130.1 (CH, ar), 127.6 (CH, ar), 127.0 (CH, ar), 124.0 (CH, ar), 86.0 (CH, d, *J*= 174.5 Hz, *C*HF), 55.4 (CH, d, *J*= 12.5 Hz, O*C*HCH_2), 40.2 (CH_2, d, *J*= 11.7 Hz, O*C*$H_2$$CH_2$), 34.0 ($CH_2$, d, *J*= 20.3 Hz, NCH*C*H_2), 30.8 (CH_2, d, *J*= 19.5 Hz, NCH_2*C*H_2), 21.6 (CH_3, arCH_3); **^{19}F, 376 MHz (δ**

in ppm): -176.0 to -176.4 (m); **HRMS m/z :** $[MNa]^+$: 401.0947, calculated 401.0947; **Mp:** 127-129 °C; **IR:** ν_{max} **(neat)/cm^{-1}:** 3043, 2941, 2873, 1598, 1519, 1493, 1346, 1159, 1094, 1015, 856.

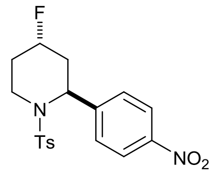

***anti*-14a**

Product ***anti*-14a** was isolated after purification on silica gel (hexane/diethyl ether, 7/3) as a colorless oil (56 mg, 30%, microwave: 46 mg, 24%).

NMR: ^{1}H, 300 MHz (δ in ppm): 8.19-8.12 (2H, m, ar), 7.77-7.71 (2H, m, ar), 7.53-7.46 (2H, m, ar), 7.36-7.29 (2H, m, ar), 5.35 (1H, d broad, *J*= 6.6 Hz, NC*H*CH_2), 4.90 (1H, dtt, *J*= 47.3 Hz, 3.8 Hz, 2.2 Hz, C*H*F), 3.85 (1H, dd, *J*= 14.6 Hz, 4.6 Hz, NC*H*HCH_2), 3.35 (1H, ddd, *J*= 14.6 Hz, 12.9 Hz, 3.1 Hz, NC*H*HCH_2), 2.75-2.66 (1H, m NCHC*H*H), 2.45 (3H, s, arCH_3), 2.01 (1H, dddd, *J*= 43.8 Hz, 15.4 Hz, 6.9 Hz, 2.4 Hz, NCHC*H*H), 1.82-1.60 (2H, m, NCH_2CH_2); **^{13}C, 100 MHz (δ in ppm):** 147.3 (Cq, ar), 146.8 (Cq, ar), 143.9 (Cq, ar), 137.6 (Cq, ar), 130.0 (CH, ar), 127.4 (CH, ar), 126.9 (CH, ar), 123.5 (CH, ar), 85.9 (CH, d, *J*= 172.6 Hz, *C*HF), 52.9 (CH, s, O*C*HCH_2), 36.6 (CH_2, s, OCH_2CH_2), 32.1 (CH_2, d, *J*= 19.7 Hz, NCHCH_2), 28.8 (CH_2, d, *J*= 21.2 Hz, NCH_2CH_2), 21.6 (CH_3, arCH_3); **^{19}F, 376 MHz (δ in ppm):** -175.7 to -176.0 (m); **HRMS m/z:** $[MNa]^+$: 401.0956, calculated 401.0947; **Mp:** 148-150 °C; **IR:** ν_{max} **(neat)/cm^{-1}:** 3081, 2940, 2851, 1598, 1519, 1494, 1345, 1158, 1091, 888.

***N*-(Tosyl)-2-pentyl-4-fluoropiperidines *syn*-14b and *anti*-14b.**

Piperidines ***syn*-14b** and ***anti*-14b** were prepared according to the general procedure outlined for the aza-Prins reaction from hexanal (61 µL, 0.5 mmol), *N*-tosyl-3-butenylamine **13** (112mg, 0.5 mmol) and boron trifluoride (65 µL, 0.5 mmol) to give the title compounds. The products were isolated as a mixture of diastereoisomer after purification on silica gel (hexane/diethyl ether, 7/3) as colorless oils (119 mg,

73%, mixture ratio *syn*/*anti* 2/1, microwave: 129 mg, 79%, mixture ratio *syn*/*anti* 1.9/1).

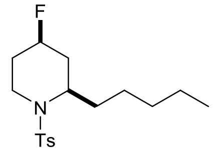

***syn*-14b**

Analysis from the mixture of diastereoisomers ***syn*-14b** and ***anti*-14b**:

NMR: ^{1}H, 300 MHz (δ in ppm): 7.77-7.68 (2H, m, ar), 7.33-7.25 (2H, m, ar), 4.72 (1H, dtt, *J*= 48.7 Hz, 11.3 Hz, 4.9 Hz, C*H*F), 4.24-4.12 (1H, m, NC*H*CH_2), 4.05-3.88 (1H, m, NC*H*HCH_2), 3.03 (1H, dddd, *J*= 14.9 Hz, 13.1 Hz, 2.4 Hz, 1.2 Hz, NC*H*HCH_2), 2.42 (3H, s, arCH_3), 2.02-1.83 (2H, m, NCHC*H*H, NCH_2C*H*H), 173-1.12 (10H, m, NCH_2C*H*H, NCHC*H*H, 4xCH_2 pentyl), 0.87 (3H, t, J= 6.7 Hz, CH_3 pentyl); **^{13}C, 75 MHz (δ in ppm):** 143.7 (Cq, ar), 138.2 (Cq, ar), 129.8 (CH, ar), 126.9 (CH, ar), 87.0 (CH, d, *J*= 173.0 Hz, *C*HF), 53.6 (CH, d, *J*= 13.0 Hz, O*C*HCH_2), 38.7 (CH_2, d, *J*= 12.3 Hz, O*C*$H_2$$CH_2$), 34.2 ($CH_2$, d, *J*= 18.2 Hz, NCH*C*H_2), 32.2 (CH_2, pentyl), 31.4 (CH_2, pentyl), 31.2 (CH_2, d, *J*= 21.7 Hz, NCH_2*C*H_2), 26.0 (CH_2, pentyl), 22.4 (CH_2, pentyl), 21.5 (CH_3, arCH_3), 13.9 (CH_3, pentyl); **^{19}F, 282 MHz (δ in ppm):** -175.8 to -176.3 (m); **HRMS m/z:** $[MNa]^+$: 350.1559, calculated 350.1566; **IR from the mixture of diastereoisomers: v_{max} (neat)/cm^{-1}:** 2952, 2918, 2851, 1457, 1331, 1300, 1200, 1144, 811.

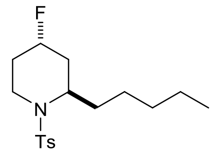

***anti*-14b**

Analysis from the mixture of diastereoisomers ***syn*-14b** and ***anti*-14b**:

NMR: ^{1}H, 300 MHz (δ in ppm): 7.77-7.68 (2H, m, ar), 7.33-7.25 (2H, m, ar), 4.83 (1H, dtt, *J*= 47.5 Hz, 3.0 Hz, 2.9 Hz, C*H*F), 4.05-3.88 (1H, m, NC*H*CH_2), 3.72 (1H, dd, *J*= 14.3 Hz, 4.9 Hz, NC*H*HCH_2), 3.31 (1H, ddd, *J*= 14.3 Hz, 13.3 Hz, 2.8 Hz, NC*H*HCH_2), 2.41 (3H, s, arCH_3), 2.02-1.83 (1H, m, NCHC*H*H), 1.83-1.73 (1H, m,

NCH_2CHH), 1.73-1.12 (10H, m, NCH_2C*H*H, NCHC*H*H, 4xCH_2 pentyl), 0.85 (3H, t, J= 6.6 Hz, CH_3 pentyl); **^{13}C, 75 MHz (δ in ppm):** 143.0 (Cq, ar), 138.5 (Cq, ar), 129.6 (CH, ar), 126.9 (CH, ar), 87.0 (CH, d, *J*= 170.0 Hz, *C*HF), 51.4 (CH, O*C*HCH$_2$), 35.0 (CH_2, O*C*H$_2$CH$_2$), 32.2 (CH_2, pentyl), 31.9 (CH_2, d, *J*= 19.2 Hz, NCH*C*H$_2$), 31.1 (CH_2, pentyl), 29.2 (CH_2, d, *J*= 21.4 Hz, NCH$_2$*C*H$_2$), 26.4 (CH_2, pentyl), 22.4 (CH_2, pentyl), 21.4 (CH_3, arCH_3), 13.9 (CH_3, pentyl); **^{19}F, 282 MHz (δ in ppm):** -179.9 to -180.7 (m); **HRMS m/z:** [MNa]$^+$: 350.1559, calculated 350.1566; **IR as a mixture of diastereoisomers: ν_{max} (neat)/cm^{-1}:** 2952, 2918, 2851, 1457, 1331, 1300, 1200, 1144, 811.

***N*-(Tosyl)-2-(4-bromophenyl)-4-fluoropiperidines *syn*-14c and *anti*-14c.**

Piperidines ***syn*-14c** and ***anti*-14c** were prepared according to the general procedure outlined for the aza-Prins reaction from 4-bromobenzaldehyde (92 mg, 0.5 mmol), *N*-tosyl-3-butenylamine **13** (112mg, 0.5 mmol) and boron trifluoride (65 µL, 0.5 mmol).

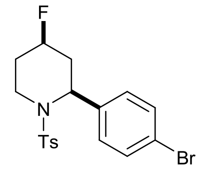

***syn*-14c**

Product ***syn*-14c** was isolated after purification on silica gel (hexane/diethyl ether, 7/3) as a white solid (72 mg, 35%, microwave: 90 mg, 44%).

NMR: ^{1}H, 400 MHz (δ in ppm): 7.78-7.68 (2H, m, ar), 7.50-7.44 (2H, m, ar), 7.37-7.31 (2H, m, ar), 7.26-7.20 (2H, m, ar), 5.35 (1H, s broad, NC*H*CH$_2$), 4.57 (1H, dtt, *J*= 48.5 Hz, 10.8 Hz, 4.5 Hz, C*H*F), 4.02-3.92 (1H, m, NC*H*HCH$_2$), 3.06-2.97 (1H, m, NC*H*HCH$_2$), 2.64-2.55 (1H, m NCHC*H*H), 2.46 (3H, s, PhCH_3), 1.92-1.83 (1H, m, NCH$_2$C*H*H), 1.76-1.65 (1H, m, NCHC*H*H), 1.44 (1H, ttd, *J*= 12.6 Hz, 10.3 Hz, 4.8 Hz, NCH$_2$C*H*H); **^{13}C, 100 MHz (δ in ppm):** 143.8 (Cq, ar), 137.6 (Cq, ar), 136.9 (Cq, ar), 131.9 (CH, ar), 130.0 (CH, ar), 128.3 (CH, ar), 126.9 (CH, ar), 121.5 (Cq, ar), 86.4 (CH, d, *J*= 174.6 Hz, *C*HF), 55.2 (CH, d, *J*= 12.4 Hz, O*C*HCH$_2$), 39.9 (CH_2, d, *J*= 12.4 Hz, O*C*H$_2$CH$_2$), 33.5 (CH_2, d, *J*= 20.2 Hz, NCH*C*H$_2$), 30.9 (CH_2, d, *J*= 18.9 Hz,

NCH_2CH_2), 21.6 (CH_3, $ArCH_3$); **^{19}F, 376 MHz (δ in ppm):** -175.2 to -175.5 (m); **HRMS m/z:** $[MNa]^+$: 434.0199, 436.0182, calculated 434.0202, 436.0181; **Mp:** 131-133 °C; **IR: ν_{max} (neat)/cm^{-1}:** 3058, 2935, 2862, 1594, 1485, 1342, 1155, 1093, 1009, 816, 668.

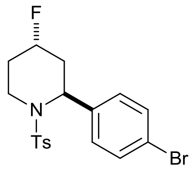

***anti*-14c**

Product ***anti*-14c** was isolated after purification on silica gel (hexane/diethyl ether, 7/3) as a white solid (68 mg, 33%, microwave : 47 mg, 23%).

NMR: ^{1}H, 300 MHz (δ in ppm): 7.74-7.67 (2H, m, ar), 7.43-7.36 (2H, m, ar), 7.32-7.26 (2H, m, ar), 7.23-7.16 (2H, m, ar), 5.19 (1H, d broad, *J*= 6.4 Hz, NC*H*CH_2), 4.87 (1H, dtt, *J*= 47.6 Hz, 3.2 Hz, 3.0 Hz, C*H*F), 3.84-3.72 (1H, m, NC*H*HCH_2), 3.36 (1H, ddd, *J*= 14.5 Hz, 12.5 Hz, 3.4 Hz, NC*H*HCH_2), 2.67-2.53 (1H, m NCHC*H*H), 2.44 (3H, s, arCH_3), 2.03 (1H, dddd, *J*= 43.0 Hz, 15.2 Hz, 6.7 Hz, 2.6 Hz, NCHC*H*H), 1.82-1.71 (1H, m, NCH_2C*H*H), 1.70-1.53 (1H, m, NCH_2C*H*H); **^{13}C, 75 MHz (δ in ppm):** 143.5 (Cq, ar), 138.5 (Cq, ar), 137.8 (Cq, ar), 131.3 (CH, ar), 129.8 (CH, ar), 128.4 (CH, ar), 126.9 (CH, ar), 120.9 (Cq, ar), 86.0 (CH, d, *J*= 171.9 Hz, *C*HF), 52.9 (CH, s, O*C*HCH_2), 36.6 (CH_2, d, *J*= 1.5 Hz, O*C*H_2CH_2), 31.9 (CH_2, d, *J*= 19.0 Hz, NCH*C*H_2), 29.1 (CH_2, d, *J*= 21.0 Hz, NCH_2*C*H_2), 21.5 (CH3, arCH_3); **^{19}F, 282 MHz (δ in ppm):** -181.2 to -181.7 (m); **HRMS m/z:** $[MH, -HF]^+$: 392.0311, 394.0311, calculated 392.0320, 394.0299; **Mp:** 121-123 °C; **IR: ν_{max} (neat)/cm^{-1}:** 3058, 2952, 2873, 1594, 1488, 1339, 1158, 1094, 1009, 814, 671.

***N*-(Tosyl)-2-(3-bromophenyl)-4-fluoropiperidines *syn*-14d and *anti*-14d.**

Piperidines ***syn*-14d** and ***anti*-14d** were prepared according to the general procedure outlined for the aza-Prins reaction from 3-bromobenzaldehyde (58 µL, 0.5 mmol), *N*-tosyl-3-butenylamine **13** (112mg, 0.5 mmol) and boron trifluoride (65 µL, 0.5 mmol).

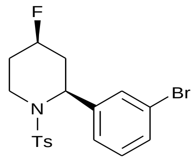

***syn*-14d**

Product ***syn*-14d** was isolated after purification on silica gel (hexane/diethyl ether, 7/3) as a white solid (82 mg, 40%).

NMR: ^{1}H, 300 MHz (δ in ppm): 7.78-7.71 (2H, m, ar), 7.40-7.28 (4H, m, ar), 7.26-7.18 (2H, m, ar), 5.38 (1H, s broad, NC*H*CH_2), 4.58 (1H, dtt, *J*= 48.5 Hz, 10.8 Hz, 4.4 Hz, C*H*F), 4.06-3.94 (1H, m, NC*H*HCH_2), 3.10-2.97 (1H, m NC*H*HCH_2), 2.65-2.52 (1H, m NCHC*H*H), 2.46 (3H, s, arCH_3), 1.95-1.82 (1H, m, NCH_2C*H*H), 1.73 (1H, dddd, *J*= 13.4 Hz, 11.1 Hz, 8.7 Hz, 5.6 Hz, NCHC*H*H), 1.44 (1H, ttd, *J*= 12.5 Hz, 10.3 Hz, 4.8 Hz, NCH_2C*H*H); **^{13}C, 75 MHz (δ in ppm):** 143.8 (Cq, ar), 140.3 (Cq, ar), 137.6 (Cq, ar), 130.6 (CH, ar), 130.4 (CH, ar), 130.0 (CH, ar), 129.5 (CH, ar), 126.9 (CH, ar), 125.2 (CH, ar), 123.1 (Cq, ar), 86.3 (CH, d, *J*= 174.4 Hz, *C*HF), 55.1 (CH, d, *J*= 12.3 Hz, O*C*HCH_2), 40.0 (CH_2, d, *J*= 12.2 Hz, O*C*$H_2$$CH_2$), 33.6 ($CH_2$, d, *J*= 19.6 Hz, NCH*C*H_2), 31.0 (CH_2, d, *J*= 19.3 Hz, NCH_2*C*H_2), 21.6 (CH_3, arCH_3); **^{19}F, 282 MHz (δ in ppm):** -175.6 to -176.0 (m); **HRMS m/z: [MNa]$^{+}$:** 434.0189, 436.0184, calculated 434.0202, 436.0181; **Mp:** 93-95 °C; **IR: ν_{max} (neat)/cm^{-1}:** 3058, 3036, 2918, 2868, 1594, 1566, 1476, 1339, 1155, 1093, 1018, 811, 741, 660.

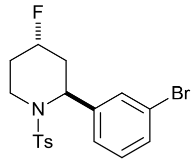

***anti*-14d**

Product ***anti*-14d** was isolated after purification on silica gel (hexane/diethyl ether, 7/3) as a white solid (40 mg, 19%).

NMR: ^{1}H, 400 MHz (δ in ppm): 7.73-7.67 (2H, m, ar), 7.35-7.29 (3H, m, ar), 7.29-7.24 (2H, m, ar), 7.18-7.13 (1H, m, ar), 5.22 (1H, d broad, *J*= 6.4 Hz, NC*H*CH_2), 4.88 (1H, dtt, *J*= 47.5 Hz, 3.5 Hz, 2.8 Hz, C*H*F), 3.85-3.76 (1H, m, NC*H*HCH_2), 3.38 (1H, ddd, *J*= 14.4 Hz, 12.6 Hz, 3.2 Hz, NC*H*HCH_2), 2.64-2.54 (1H, m NCHC*H*H), 2.44

(3H, s, $PhCH_3$), 1.98 (1H, dddd, *J*= 43.1 Hz, 15.1 Hz, 6.8 Hz, 2.6 Hz, NCHC*H*H), 1.86-1.76 (1H, m, NCH_2C*H*H), 1.75-1.61 (1H, m, NCH_2C*H*H); **^{13}C, 100 MHz (δ in ppm):** 143.7 (Cq, ar), 142.0 (Cq, ar), 137.8 (Cq, ar), 130.0 (CH, ar), 129.9 (CH, ar), 129.8 (CH, ar), 129.7 (CH, ar), 126.9 (CH, ar), 125.3(CH, ar), 122.5 (Cq, ar), 86.0 (CH, d, *J*= 172.2 Hz, *C*HF), 52.9 (CH, s, O*C*HCH_2), 36.8 (CH_2, s, O*C*H_2CH_2), 32.3 (CH_2, d, *J*= 19.3 Hz, NCH*C*H_2), 29.2 (CH_2, d, *J*= 21.2 Hz, NCH_2*C*H_2), 21.6 (CH3, $ArCH_3$); **^{19}F, 376 MHz (δ in ppm):** -181.4 to -181.9 (m); **HRMS m/z:** $[MNa]^+$: 434.0201, 436.0183, calculated 434.0202, 436.0181; **Mp:** 105-107 °C; **IR: ν_{max} (neat)/cm^{-1}:** 3057, 2974, 2865, 1594, 1563, 1474, 1325, 1281, 1130, 1068, 886, 746, 710, 648.

***N*-(Tosyl)-2-(2-bromophenyl)-4-fluoropiperidines *syn*-14e and *anti*-14e.**

Piperidines ***syn*-14e** and ***anti*-14e** were prepared according to the general procedure outlined for the aza-Prins reaction from 2-bromobenzaldehyde (58 µL, 0.5 mmol), *N*-tosyl-3-butenylamine **13** (112mg, 0.5 mmol) and boron trifluoride (65 µL, 0.5 mmol).

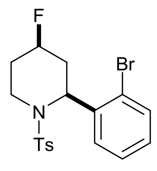

***syn*-14e**

Product ***syn*-14e** was isolated after purification on silica gel (hexane/diethyl ether, 7/3) as a colorless oil (51 mg, 25%).

NMR: ^{1}H, 300 MHz (δ in ppm): 7.68-7.63 (2H, m, ar), 7.44 (1H, dd, *J*= 8.0 Hz, 1.5 Hz, ar), 7.36 (1H, dd, *J*= 8.0 Hz, 2.0 Hz, ar), 7.21-7.16 (3H, m, ar), 7.07-7.01 (1H, m, ar), 5.14 (1H, t, *J*= 6.0 Hz, NC*H*CH_2), 4.78 (1H, dtt, *J*= 48.5 Hz, 6.2 Hz, 3.8 Hz, C*H*F), 3.97-3.87 (1H, m, NC*H*HCH_2), 3.71 (1H, dt, *J*= 13.9 Hz, 5.1 Hz, NC*H*HCH_2), 2.41-2.29 (4H, s, NCHC*H*H, $arCH_3$), 2.18-2.05 (1H, m, NCHC*H*H), 2.01-1.82 (2H, NCH_2*C*H_2); **^{13}C, 100 MHz (δ in ppm):** 143.2 (Cq, ar), 140.3 (Cq, ar), 136.4 (Cq, ar), 133.1 (CH, ar), 129.5 (CH, ar), 129.4 (CH, ar), 127.3 (CH, ar), 127.3 (CH, ar), 127.2 (CH, ar), 122.0 (Cq, ar), 86.6 (CH, d, *J*= 173.8 Hz, *C*HF), 54.8 (CH, d, *J*= 6.3 Hz, O*C*HCH_2), 40.4 (CH_2, d, *J*= 6.3 Hz, O*C*H_2CH_2), 35.2 (CH_2, d, *J*= 20.2 Hz, NCH*C*H_2),

30.0 (CH_2, d, *J*= 20.8 Hz, NCH_2CH_2), 21.4 (CH_3, arCH_3); **^{19}F, 282 MHz (δ in ppm):** -175.6 to -176.0 (m); **HRMS m/z:** [MNa, -HF]$^+$: 414.0145, 416.0103, calculated 414.0139, 416.0119; **IR: ν_{max} (neat)/cm^{-1}:** 3058, 3025, 2918, 2840, 1594, 1563, 1460, 1328, 1158, 1090, 1020, 811, 749, 704, 648.

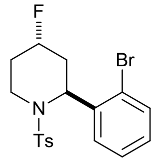

***anti*-14e**

Product ***anti*-14e** was isolated after purification on silica gel (hexane/diethyl ether, 7/3) as a colorless oil (49 mg, 24%).

NMR: ^{1}H, 400 MHz (δ in ppm): 7.53-7.48 (2H, m, ar), 7.42 (1H, dd, *J*= 8.0 Hz, 1.2 Hz, ar), 7.37 (1H, dd, *J*= 7.8 Hz, 1.6 Hz, ar), 7.24-7.16 (3H, m, ar), 7.07 (1H, td, *J*= 7.6 Hz, 1.6 Hz, ar), 5.21 (1H, dd, *J*= 7.7 Hz, 5.1 Hz, NC*H*CH_2), 4.81 (1H, dtt, *J*= 48.5 Hz, 6.4 Hz, 3.7 Hz, C*H*F), 3.81-3.70 (2H, m, NC$H_2$$CH_2$), 2.44-2.29 (4H, m NCHC*H*H, arCH_3), 2.19-1.99 (2H, m, NCHC*H*H, NCH_2C*H*H), 1.92-1.77 (1H, m, NCH_2C*H*H); **^{13}C, 100 MHz (δ in ppm):** 143.2 (Cq, ar), 139.4 (Cq, ar), 135.9 (Cq, ar), 133.1 (CH, ar), 129.3 (CH, ar), 128.8 (CH, ar), 128.8 (CH, ar), 127.3 (CH, ar), 127.2 (CH, ar), 122.9 (Cq, ar), 86.3 (CH, d, *J*= 172.4 Hz, *C*HF), 55.8 (CH, d, *J*= 6.7 Hz, O*C*HCH_2), 42.5 (CH_2, d, *J*= 6.3 Hz, OCH_2CH_2), 35.8 (CH_2, d, *J*= 20.7 Hz, NCHCH_2), 30.6 (CH_2, d, *J*= 20.7 Hz, NCH_2CH_2), 21.5 (CH_3, arCH_3); **^{19}F, 376 MHz (δ in ppm):** -180.9 to -181.4 (m) ; **HRMS m/z: [MNa]$^+$:** 434.0195, 436.0186, calculated 434.0202, 436.0181; **IR: ν_{max} (neat)/cm^{-1}:** 3022, 2985, 2845, 1558, 1281, 1180, 1124, 1057, 892, 760, 671.

***N*-(Tosyl)-2-(4-fluorophenyl)-4-fluoropiperidines *syn*-14f and *anti*-14f.**

Piperidines ***syn*-14f** and ***anti*-14f** were prepared according to the general procedure outlined for the aza-Prins reaction from 4-fluorobenzaldehyde (53 μL, 0.5 mmol), *N*-tosyl-3-butenylamine **13** (112mg, 0.5 mmol) and boron trifluoride (65 μL, 0.5 mmol).

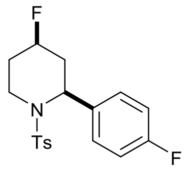

***syn*-14f**

Product ***syn*-14f** was isolated after purification on silica gel (hexane/diethyl ether, 7/3) as a white solid (78 mg, 44%, microwave: 66 mg, 38%).

NMR: ^{1}H, 400 MHz (δ in ppm): 7.80-7.73 (2H, m, ar), 7.37-7.30 (4H, m, ar), 7.07-7.01 (2H, m, ar), 5.38 (1H, s broad, NC*H*CH$_2$), 4.60 (1H, dtt, *J*= 48.5 Hz, 10.9 Hz, 4.5 Hz, C*H*F), 4.03-3.93 (1H, m, NC*H*HCH$_2$), 3.03 (1H, dddd, *J*= 14.7 Hz, 12.7 Hz, 2.6 Hz, 1.3 Hz, NC*H*HCH$_2$), 2.66-2.56 (1H, m NCHC*H*H), 2.46 (3H, s, PhCH$_3$), 1.92-1.83 (1H, m, NCH$_2$C*H*H), 1.70 (1H, dddd, *J*= 13.4 Hz, 11.0 Hz, 8.7 Hz, 5.5 Hz, NCHC*H*H), 1.44 (1H, ttd, *J*= 12.6 Hz, 10.3 Hz, 4.8 Hz, NCH$_2$C*H*H); **^{13}C, 100 MHz (δ in ppm):** 162.0 (Cq, d, *J*= 249.8 Hz, ar), 143.7 (Cq, ar), 137.8 (Cq, ar), 134.5 (Cq, ar), 130.0 (CH, ar), 128.3 (CH, d, *J*= 8.0 Hz, ar), 126.9 (CH, ar), 115.7 (CH, d, *J*= 21.2 Hz, ar), 86.5 (CH, d, *J*= 173.1 Hz, *C*HF), 55.1 (CH, d, *J*= 13.1 Hz, O*C*HCH$_2$), 39.9 (CH$_2$, d, *J*= 13.5 Hz, O*C*H$_2$CH$_2$), 33.6 (CH$_2$, d, *J*= 19.6 Hz, NCH*C*H$_2$), 31.0 (CH$_2$, d, *J*= 19.6 Hz, NCH$_2$*C*H$_2$), 21.6 (CH$_3$, ArCH$_3$); **^{19}F, 376 MHz (δ in ppm):** -115.6 to -115.7 (m), -175.5 to -176.0 (m); **HRMS m/z:** [MNa]$^+$: 374.1001, calculated 374.1002; **Mp:** 111-113 °C; **IR: v_{max} (neat)/cm^{-1}:** 3043, 2941, 2873, 1597, 1507, 1339, 1151, 1093, 838.

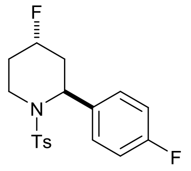

***anti*-14f**

The product ***anti*-14f** was isolated after purification on silica gel (hexane/diethyl ether, 7/3) as a colorless oil (42 mg, 24%, microwave: 44 mg, 25%).

NMR: ^{1}H, 400 MHz (δ in ppm): 7.75-7.67 (2H, m, ar), 7.35-7.25 (4H, m, ar), 7.02-6.91 (2H, m, ar), 5.22 (1H, d broad, *J*= 6.3 Hz, NC*H*CH$_2$), 4.87 (1H, dtt, *J*= 47.6 Hz, 3.4 Hz, 3.0 Hz, C*H*F), 3.83-3.72 (1H, m, NC*H*HCH$_2$), 3.37 (1H, ddd, *J*= 14.4 Hz, 12.6 Hz, 3.0 Hz, NC*H*HCH$_2$), 2.66-2.55 (1H, m NCHC*H*H), 2.44 (3H, s, PhCH$_3$), 1.96 (1H,

dddd, J= 43.2 Hz, 15.3 Hz, 6.8 Hz, 2.7 Hz, NCHC*H*H), 1.81-1.70 (1H, m, NCH_2C*H*H), 1.70-1.55 (1H, m, NCH_2C*H*H); **^{13}C, 100 MHz (δ in ppm):** 161.7 (Cq, d, J= 245.6 Hz), 143.4 (Cq, ar), 137.9 (Cq, ar), 135.1 (Cq, d, J= 2.9 Hz, ar), 129.8 (CH, ar), 128.3 (CH, dd, J= 8.0 Hz, 2.5 Hz, ar), 126.9 (CH, ar), 115.0 (CH, d, J= 21.3 Hz, ar), 86.1 (CH, d, J= 172.4 Hz, *C*HF), 52.9 (CH, s, O*C*HCH_2), 36.6 (CH_2, OCH_2CH_2), 32.0 (CH_2, d, J= 19.3 Hz, NCHCH_2), 29.1 (CH_2, d, J= 21.1 Hz, NCH_2CH_2), 21.5 (CH3, ArCH_3); **^{19}F, 376 MHz (δ in ppm):** -116.7 to 116.8 (m), -180.8 to -181.3 (m); **HRMS m/z: [MNa]$^+$:** 374.1001, calculated 374.1002; **Mp:** 127-129 °C; **IR: v_{max} (neat)/cm^{-1}:** 3043, 2920, 2873, 1597, 1508,1328, 1152, 1071, 1032, 882.

N-(Tosyl)-2-(3-fluorophenyl)-4-fluoropiperidines *syn*-14g and *anti*-14g.

Piperidines ***syn*-14g** and ***anti*-14g** were prepared according to the general procedure outlined for the aza-Prins reaction from 3-fluorobenzaldehyde (55 µL, 0.5 mmol), *N*-tosyl-3-butenylamine **13** (112mg, 0.5 mmol) and boron trifluoride (65 µL, 0.5 mmol).

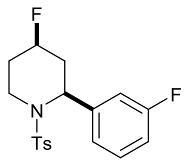

***syn*-14g**

Product ***syn*-14g** was isolated after purification on silica gel (hexane/diethyl ether, 7/3) as a white solid (78 mg, 44%).

NMR: ^{1}H, 300 MHz (δ in ppm): 7.81-7.74 (2H, m, ar), 7.39-7.32 (2H, m, ar), 7.30-7.22 (1H, m, ar), 7.18-7.11 (1H, m, ar), 7.09-7.01 (1H, m, ar), 7.01-6.92 (1H, m, ar), 5.41 (1H, s broad, NC*H*CH_2), 4.57 (1H, dtt, J= 48.5 Hz, 10.9 Hz, 4.5 Hz, C*H*F), 4.07-3.94 (1H, m, NC*H*HCH_2), 3.03 (1H, dddd, J= 14.9 Hz, 12.8 Hz, 2.9 Hz, 1.2 Hz, NC*H*HCH_2), 2.67-2.55 (1H, m NCHC*H*H), 2.46 (3H, s, PhCH_3), 1.94-1.82 (1H, m, NCH_2C*H*H), 1.72 (1H, dddd, J= 13.4 Hz, 11.2 Hz, 8.6 Hz, 5.7 Hz, NCHC*H*H), 1.45 (1H, ttd, J= 12.7 Hz, 10.3 Hz, 4.9 Hz, NCH_2C*H*H); **^{13}C, 75 MHz (δ in ppm):** 163.2 (Cq, d, J= 247.7 Hz, *C*HF), 143.7 (Cq, ar), 140.7 (Cq, d, J= 7.7 Hz, ar), 137.6 (Cq, ar), 130.4 (CH, d, J= 8.7 Hz, ar), 130.0 (CH, ar), 129.5 (CH, d, J= 8.7 Hz, ar), 126.9 (CH, ar), 114.4 (CH, t, J= 21.3 Hz, ar), 113.6 (CH, d, J= 22.7 Hz, ar), 86.4 (CH, d, J=

174.9 Hz, *C*HF), 55.2 (CH, d, *J*= 12.5 Hz, O*C*HCH$_2$), 40.0 (CH$_2$, d, *J*= 11.7 Hz, O*C*H$_2$CH$_2$), 33.6 (CH$_2$, d, *J*= 20.5 Hz, NCH*C*H$_2$), 30.9 (CH$_2$, d, *J*= 19.0 Hz, NCH$_2$*C*H$_2$), 21.5 (CH$_3$, ar*C*H$_3$); **^{19}F, 376 MHz (δ in ppm):** -112.0 to -112.1 (m), -175.0- -175.3 (m); **HRMS m/z:** [MNa]$^+$: 374.1002, calculated 374.1002; **Mp:** 101-103 °C; **IR: v_{max} (neat)/cm^{-1}:** 3065, 2944, 2877, 1614, 1590, 1488, 1341, 1160, 1096, 1019, 889, 736, 674.

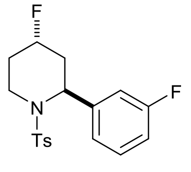

***anti*-14g**

Product ***anti*-14g** was isolated after purification on silica gel (hexane/diethyl ether, 7/3) as a white solid (39 mg, 22%).

NMR: ^{1}H, 300 MHz (δ in ppm): 7.70-7.69 (2H, m, ar), 7.35-7.28 (2H, m, ar), 7.24 (1H, dd, *J*= 8.0 Hz, 6.0 Hz, ar), 7.14-7.08 (1H, m, ar), 7.04-6.96 (1H, m, ar), 6.95-6.85 (1H, m, ar)5.26 (1H, d broad, *J*= 6.6 Hz, NC*H*CH$_2$), 4.88 (1H, dtt, *J*= 47.3 Hz, 3.2 Hz, 2.8 Hz, C*H*F), 3.86-3.75 (1H, m, NC*H*HCH$_2$), 3.38 (1H, ddd, *J*= 14.4 Hz, 12.6 Hz, 3.3 Hz, NC*H*HCH$_2$), 2.68-2.55 (1H, m, NCHC*H*H), 2.44- (3H, s, arCH$_3$), 1.98 (1H, dddd, *J*= 43.6 Hz, 15.2 Hz, 6.9 Hz, 2.6 Hz, NCHC*H*H), 1.82-1.72 (1H, m, NCH$_2$C*H*H), 1.71-1.53 (1H, m, NCH$_2$C*H*H); **^{13}C, 75 MHz (δ in ppm):** 162.8 (Cq, d, *J*= 246.2 Hz, *C*HF), 143.5 (Cq, ar), 142.3 (Cq, d, *J*= 6.7 Hz, ar), 137.8 (Cq, ar), 129.8 (CH, ar), 129.6 (CH, d, *J*= 8.3 Hz, ar), 126.9 (CH, ar), 122.1 (CH, t, *J*= 2.3 Hz, ar), 113.7 (CH, d, *J*= 21.0 Hz, ar), 113.6 (CH, dd, *J*= 2.9 Hz, 2.4 Hz, ar), 85.9 (CH, d, *J*= 172.2 Hz, *C*HF), 52.8 (CH, d, *J*= 6.7 Hz, O*C*HCH$_2$), 36.6 (CH$_2$, O*C*H$_2$CH$_2$), 32.0 (CH$_2$, d, *J*= 20.4 Hz, NCH*C*H$_2$), 29.0 (CH$_2$, d, *J*= 21.0 Hz, NCH$_2$*C*H$_2$), 21.5 (CH$_3$, ar*C*H$_3$); **^{19}F, 376 MHz (δ in ppm):** -113.4 to 113.5 (m), -186.0 to -186.5 (m) ; **HRMS m/z:** [MNa]$^+$: 374.1013, calculated 374.1002; **Mp:** 111-113 °C; **IR: v_{max} (neat)/cm^{-1}:** 2974, 2913, 2845, 1611, 1588, 1348, 1269, 1186, 1127, 1068, 903, 732, 654.

N-(Tosyl)-2-(4-methoxyphenyl)-4-fluoropiperidines *syn*-14h and *anti*-14h.

Piperidines ***syn*-14h** and ***anti*-14h** were prepared according to the general procedure outlined for the aza-Prins reaction from 4-methoxybenzaldehyde (60 µL, 0.5 mmol), *N*-tosyl-3-butenylamine **13** (112mg, 0.5 mmol) and boron trifluoride (65 µL, 0.5 mmol).

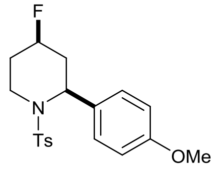

***syn*-14h**

Product ***syn*-14h** was isolated after purification on silica gel (hexane/diethyl ether, 7/3) as a pale yellow viscous oil (26 mg, 14%).

NMR: ^{1}H, 400 MHz (δ in ppm): 7.80-7.74 (2H, m, ar), 7.37-7.31 (2H, m, ar), 7.30-7.23 (2H, m, ar), 6.90-6.85 (2H, m, ar), 5.38 (1H, s broad, NC*H*CH_2), 4.64 (1H, dtt, *J*= 48.7 Hz, 10.9 Hz, 4.4 Hz, C*H*F), 4.02-3.91 (1H, m, NC*H*HCH_2), 3.81 (3H, s, OCH_3), 3.02 (1H, dddd, *J*= 14.9 Hz, 12.7 Hz, 2.6 Hz, 1.0 Hz, NC*H*HCH_2), 2.67-2.58 (1H, m NCHC*H*H), 2.46 (3H, s, arCH_3), 1.91-1.81 (1H, m, NCH_2C*H*H), 1.68 (1H, dddd, *J*= 13.2 Hz, 11.2 Hz, 9.0 Hz, 5.7 Hz, NCHC*H*H), 1.43 (1H, ttd, *J*= 12.5 Hz, 10.5 Hz, 4.9 Hz, NCH_2C*H*H); **^{13}C, 100 MHz (δ in ppm):** 158.8 (Cq, ar), 143.5 (Cq, ar), 138.0 (Cq, ar), 129.9 (CH, ar), 129.5 (Cq, ar), 127.7 (CH, ar), 126.9 (CH, ar), 114.2 (CH, ar), 86.8 (CH, d, *J*= 176.8 Hz, *C*HF), 55.3 (CH_3, OCH_3), 55.1 (CH, d, *J*= 12.9 Hz, O*C*HCH_2), 39.8 (CH_2, d, *J*= 12.5 Hz, O*C*H_2CH_2), 33.5 (CH_2, d, *J*= 19.6 Hz, NCH*C*H_2), 31.1 (CH_2, d, *J*= 18.3 Hz, NCH_2*C*H_2), 21.5 (CH_3, arCH_3); **^{19}F, 376 MHz (δ in ppm):** -175.5 to -175.8 (m); **HRMS m/z:** $[MNa]^+$: 386.1201, calculated 386.1202; **IR of the mixture of diastereoisomers: ν_{max} (neat)/cm^{-1}:** 2974, 2918, 2845, 1510, 1348, 1303, 1180, 1144, 1012, 881.

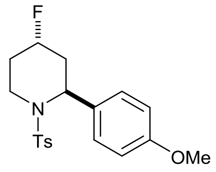

***anti*-14h**

Product ***anti*-14h** was isolated as a mixture of diastereoisomers (*syn*/*anti,* 1/2.2) after purification on silica gel (hexane/diethyl ether, 7/3) as a pale yellow viscous oil (13 mg, 7%).

NMR: ^{1}H, 400 MHz (δ in ppm): 7.74-7.67 (2H, m, ar), 7.30-7.21 (4H, m, ar), 6.84-6.77 (2H, m, ar), 5.19 (1H, d broad, *J*= 6.2 Hz, NC*H*CH_2), 4.87 (1H, dtt, *J*= 47.8 Hz, 3.6 Hz, 2.7 Hz, C*H*F), 3.79 (3H, s, OCH_3), 3.77-3.70 (1H, m, NC*H*HCH_2), 3.40 (1H, ddd, *J*= 14.5 Hz, 12.2 Hz, 3.4 Hz, NC*H*HCH_2), 2.66-2.55 (1H, m NCHC*H*H), 2.43 (3H, s, arCH_3), 1.97 (1H, dddd, *J*= 42.6 Hz, 15.2 Hz, 6.8 Hz, 2.8 Hz, NCHC*H*H), 1.82-1.74 (1H, m, NCH_2C*H*H), 1.70-1.64 (1H, m, NCH_2C*H*H); **^{13}C, 100 MHz (δ in ppm):** 148.6 (Cq, ar), 143.3 (Cq, ar), 138.0 (Cq, ar), 131.3 (Cq, ar), 129.7 (CH, ar), 127.9 (CH, ar), 127.0 (CH, ar), 113.5 (CH, ar), 86.3 (CH, d, *J*= 173.7 Hz, *C*HF), 55.2 (CH_3, OCH_3), 53.2 (CH, s, O*C*HCH_2), 36.7 (CH_2, OCH_2CH_2), 32.1 (CH_2, d, *J*= 19.9 Hz, NCH*C*H_2), 29.1 (CH_2, d, *J*= 19.6 Hz, NCH_2*C*H_2), 21.5 (CH_3, arCH_3); **^{19}F, 376 MHz (δ in ppm):** -180.1 to -180.6 (m); **HRMS m/z**: $[MNa]^+$: 386.1208, calculated 386.1202; **IR of the mixture of diastereoisomers: v_{max} (neat)/cm^{-1}:** 2952, 2918, 2845, 1608, 1591, 1337, 1303, 1146, 1090, 833.

***N*-(Tosyl)-2-methyl-4-fluoropiperidines *syn*-14j and *anti*-14j.**

Piperidines ***syn*-14j** and ***anti*-14j** were prepared according to the general procedure outlined for the aza-Prins reaction from acetaldehyde (28 µL, 0.5 mmol), *N*-tosyl-3-butenylamine **13** (112mg, 0.5 mmol) and boron trifluoride (65 µL, 0.5 mmol) to give the title compounds (99 mg, 73%, mixture ratio *syn*/*anti* 1.2/1, microwave: 104 mg, 77%, mixture ratio *syn*/*anti* 1.2/1).

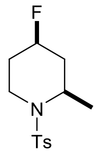

***syn*-14j**

Analysis from the mixture of diastereoisomers ***syn*-14j** and ***anti*-14j**:

NMR: ^{1}H, 300 MHz (δ in ppm): 7.75-7.66 (2H, m, ar), 7.34-7.24 (2H, m, ar), 4.77 (1H, dtt, *J*= 48.5 Hz, 10.9 Hz, 4.7 Hz, C*H*F), 4.42-4.29 (1H, m, NC*H*CH$_2$), 3.94-3.79 (1H, m, NC*H*HCH$_2$), 3.10 (1H, dddd, *J*= 13.9 Hz, 12.7 Hz, 2.7 Hz, 1.2 Hz, NC*H*HCH$_2$), 2.42 (3H, s, arCH$_3$), 2.08-1.96 (1H, m NCH$_2$C*H*H), 1.95-1.45 (3H, m, NCH$_2$C*H*H, NCHC*H$_2$*), 1.12, (3H, d, J= 7.1 Hz, CHC*H$_3$*); **^{13}C, 75 MHz (δ in ppm):** 143.3 (Cq, ar), 137.6 (Cq, ar), 129.7 (CH, ar), 126.8 (CH, ar), 86.7 (CH, d, *J*= 173.0 Hz, *C*HF), 49.0 (CH, d, *J*= 12.8 Hz, O*C*HCH$_2$), 38.6 (CH$_2$, d, *J*= 12.5 Hz, O*C*H$_2$CH$_2$), 36.7 (CH$_2$, d, *J*= 18.6 Hz, NCH*C*H$_2$), 31.7 (CH$_2$, d, *J*= 19.1 Hz, N*C*H$_2$CH$_2$), 21.4 (CH$_3$, arCH$_3$), 17.0 (CH$_3$, CH*C*H$_3$); **^{19}F, 282 MHz (δ in ppm):** -179.8 to -180.4 (m); **HRMS m/z:** [MNa]$^+$: 294.0942, calculated 294.0940; **IR from the mixture of diastereoisomers: v_{max} (neat)/cm^{-1}:** 3058, 3030, 2941, 2873, 1594, 1348, 1331, 1160, 1001, 813.

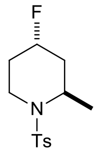

***anti*-14j**

Analysis from the mixture of diastereoisomers ***syn*-14j** and ***anti*-14j**:

NMR: ^{1}H, 300 MHz (δ in ppm): 7.75-7.66 (2H, m, ar), 7.34-7.24 (2H, m, ar), 4.88 (1H, dtt, *J*= 47.3 Hz, 3.1 Hz, 2.9 Hz, C*H*F), 4.29-4.16 (1H, m, NC*H*CH$_2$), 3.70 (1H, dd broad, *J*= 13.7 Hz; 5.3 Hz, NC*H*HCH$_2$), 3.31 (1H, td, *J*= 13.1 Hz, 2.7 Hz, NC*H*HCH$_2$), 2.41 (3H, s, arCH$_3$), 1.95-1.45 (4H, m, NCHC*H$_2$*, NCH$_2$C*H$_2$*), 1.17 (3H, dd, *J*= 7.2 Hz, 1.8 Hz, CHCH$_3$); **^{13}C, 75 MHz (δ in ppm):** 143.1 (Cq, ar), 138.0 (Cq, ar), 129.6 (CH, ar), 126.8 (CH, ar), 86.8 (CH, d, *J*= 170.3 Hz, *C*HF), 46.9 (CH, O*C*HCH$_2$), 34.7 (CH$_2$, O*C*H$_2$CH$_2$), 34.6 (CH$_2$, d, *J*= 18.9 Hz, NCH*C*H$_2$), 29.8 (CH$_2$, d, *J*= 21.4 Hz, NCH$_2$*C*H$_2$), 21.4 (CH$_3$, arCH$_3$), 17.9 (CH$_3$ CH*C*H$_3$); **^{19}F, 282 MHz (δ in ppm):** -179.8 to -180.4 (m); **HRMS m/z:** [MNa]$^+$: 294.0942, calculated 294.0940; **IR from a**

mixture of diastereoisomers: v_{max} (neat)/cm^{-1}: 3058, 3030, 2941, 2873, 1594, 1348, 1331, 1160, 1001, 813.

***N*-(Tosyl)-2-isobutyl-4-fluoropiperidines *syn*-14k and *anti*-14k.**

Piperidines ***syn*-14k** and ***anti*-14k** were prepared according to the general procedure outlined for the aza-Prins reaction from isobutyraldehyde (45 µL, 0.5 mmol), *N*-tosyl-3-butenylamine **13** (112mg, 0.5 mmol) and boron trifluoride (65 µL, 0.5 mmol). The product was isolated as a mixture of diastereoisomers after purification on silica gel (hexane/diethyl ether, 7/3) as a pale yellow oil (111 mg, 82%, mixture ratio *syn*/*anti* 1/1, microwave: 112 mg, 83%, mixture ratio *syn*/*anti* 1.2/1).

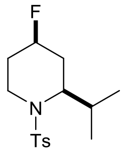

***syn*-14k**

Analysis from the mixture of diastereoisomers ***syn*-14k** and ***anti*-14k**:

NMR: ^{1}H, 300 MHz (δ in ppm): 7.78-7.71 (2H, m, ar), 7.34-7.28 (2H, m, ar), 4.67 (1H, dtt, *J*= 48.8 Hz, 11.3 Hz, 4.6 Hz, C*H*F), 4.04-3.92 (1H, m, NC*H*HCH$_2$), 3.80-3.71(1H, m, NC*H*CH$_2$), 2.99 (1H, dddd, *J*= 15.3 Hz, 13.6 Hz, 2.7 Hz, 1.3 Hz, NC*H*HCH$_2$), 2.44 (3H, s, arCH$_3$), 2.21-2.10 (1H, m NCHC*H*H), 1.93-1.75 (2H, m, NCH$_2$C*H*H, C*H*(CH$_3$)$_2$), 1.37-1.22 (2H, m, NCH$_2$C*H*H, NCHC*H*H), 0.95 (3H, d, J= 6.8 Hz, CHC*H$_3$*), 0.81 (3H, d, *J*= 6.77 Hz, CHC*H$_3$*); **^{13}C, 75 MHz (δ in ppm):** 143.3 (Cq, ar), 138.4 (Cq, ar), 129.8 (CH, ar), 126.9 (CH, ar), 86.9 (CH, d, *J*= 172.2 Hz, *C*HF), 60.1 (CH, d, *J*= 12.3 Hz, O*C*HCH$_2$), 39.1 (CH$_2$, d, *J*= 12.5 Hz, O*C*H$_2$CH$_2$), 31.6 (CH$_2$, d, *J*= 17.7 Hz, NCH*C*H$_2$), 30.8 (CH$_2$, d, *J*= 19.2 Hz, N*C*H$_2$CH$_2$), 27.7 (CH, *C*H(CH$_3$)$_2$), 21.5 (CH$_3$, CH*C*H$_3$), 20.2 (CH$_3$, CH*C*H$_3$), 19.9 (CH$_3$, ar*C*H$_3$); **^{19}F, 282 MHz (δ in ppm):** -174.4 to -174.9 (m); **HRMS m/z:** [MNa]$^{+}$: 322.1256, calculated 322.1253; **IR from the mixture of diastereoisomers: v_{max} (neat)/cm^{-1}:** 2969, 2918, 2868, 1454, 1337, 1303, 1208, 1149, 1090, 818.

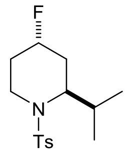

***anti*-14k**

Analysis from the mixture of diastereoisomers ***syn*-14k** and ***anti*-14k**:

NMR: ^{1}H, 300 MHz (δ in ppm): 7.77-7.70 (2H, m, ar), 7.35-7.28 (2H, m, ar), 4.79 (1H, dtt, *J*= 48.3 Hz, 2.9 Hz, 2.6 Hz, C*H*F), 3.80-3.67 (1H, NC*H*HCH$_2$), 3.54 (1H, dd, *J*= 10.9 Hz, 6.2 Hz, NC*H*CH$_2$), 3.29 (1H, ddd, *J*= 15.0 Hz, 13.4 Hz, 3.0 Hz, NC*H*HCH$_2$), 2.41 (3H, s, arCH$_3$), 2.29-2.08 (2H, m, NCHC*H*H, C*H*(CH$_3$)$_2$), 1.74-1.34 (3H, m, NCH$_2$C*H*H, NCH$_2$C*H*H, NCHC*H*H), 0.94 (3H, d, J= 6.5 Hz, CHC*H$_3$*), 0.85 (3H, d, *J*= 6.6 Hz, CHC*H$_3$*); **^{13}C, 75 MHz (δ in ppm):** 143.0 (Cq, ar), 138.6 (Cq, ar), 129.6 (CH, ar), 126.9 (CH, ar), 87.2 (CH, d, *J*= 170.0 Hz, *C*HF), 57.9 (CH, O*C*HCH$_2$), 35.5 (CH$_2$, O*C*H$_2$CH$_2$), 29.1 (CH$_2$, d, *J*= 19.8 Hz, NCH*C*H$_2$), 28.9 (CH, *C*H(CH$_3$)$_2$), 28.7 (CH$_2$, d, *J*= 20.7 Hz, NCH$_2$*C*H$_2$), 21.5 (CH$_3$, arCH$_3$), 20.7 (CH$_3$ CH*C*H$_3$), 20.1 (CH$_3$ CH*C*H$_3$); **^{19}F, 282 MHz (δ in ppm):** -181.4 to -182.2 (m); **HRMS m/z:** [MNa]$^+$: 322.1256, calculated 322.1253; **IR: ν_{max} (neat)/cm^{-1}:** 2969, 2918, 2868, 1454, 1337, 1303, 1208, 1149, 1090, 818.
